# Impact Analysis of the Market Penetration Rate of Connected Vehicles and the Failure Rate of Roadside Equipment on Data Accuracy

**DOI:** 10.3390/s26020686

**Published:** 2026-01-20

**Authors:** Fengping Zhan

**Affiliations:** School of Automotive and Traffic Engineering, Jiangsu University, Zhenjiang 212013, China; fpzhan@ujs.edu.cn

**Keywords:** data accuracy, market penetration rate (MPR), sensor failure rate, optimal deployment method, data processing algorithm

## Abstract

**Highlights:**

**What are the main findings?**

- The optimal deployment method solved by the Simulated Annealing Genetic Algorithm (SAGA) outperforms the SA algorithm and GA, which are superior to the uniform method and the hotspot method in optimizing RSE locations for improving data accuracy.
- The accuracy of single-source data can be improved along with the increase in CV MPR but decreases with the increase in sensor failure rate. The fused data is less affected by the failure rates. Multi-source data fusion is much more effective in improving data accuracy than missing data imputation. When the MPR is higher than 15% or the failure rate exceeds 40%, it is recommended to adopt data fusion rather than repairing missing data.

**What are the implications of the main findings?**

- It provides a method that is more suitable to address the equipment optimization issue at the road segment level and can better balance the spatial selection fairness of sensor locations.
- The findings shed light on the trade-offs and benefits associated with improving RSE deployment and promoting CV development.

**Abstract:**

Data quality, involving the accuracy, completeness and reliability of data, is of great significance for the operation and management of road traffic. As the two significant factors that affect data accuracy, the market penetration rate (MPR) of CVs and the failure rate of roadside equipment (RSE) were considered in the heterogeneity traffic flow comprising human-driven vehicles and CVs. An optimal deployment method solved by SAGA was proposed to optimize the locations of RSE. A rigid nearest neighbor (RNN) algorithm and a soft nearest neighbor (SNN) algorithm were addressed to handle the missing data caused by sensor failure. Additionally, the BPNN algorithm was adopted to fuse RSE data and CV data. Case analysis results show that the proposed optimal deployment method is superior to the uniform and the hotspot methods. Data accuracy can reach 95% and 98% when the MPR is 15% and 60%, respectively. It decreases with the increase in sensor failure rate for single-source data, but not for the fused data. The performance of the SNN algorithm is better than the RNN algorithm in fixing single-source missing data. However, multi-source data fusion, especially with the high-precision data, is much more effective in improving data accuracy than missing data imputation.

## 1. Introduction

In the foreseeable future, connected vehicles (CVs) will coexist with human-driven vehicles resulting in a heterogeneity traffic flow. There would be a prolonged transition period towards full CV deployment, during which not all vehicles are connected and the complete information of the traffic is typically unavailable [1]. A connected vehicle is the vehicle that uses several different communication technologies to communicate with the drive, other vehicles on the road (V2V), roadside infrastructure (V2I), and the “Cloud” (V2C). V2I technology can enable vehicle probe data applications, providing detailed traffic information such as speed, volume, travel time, queue length, and stops [2]. The CV market penetration rate (MPR), which is generally defined as the ratio of the number of CVs to the number of all traffic traveling on the road over a period, is the fundamental component of tremendous applications (such as the traffic state estimation) [3]. To investigate operational or safety effects of CVs, it is essential to take MPR into consideration because the performance of CVs is significantly impacted by the MPR [4]. However, higher MPR is not guaranteed to yield greater benefits in a transportation system, especially in cases where the related technologies, facilities, and costs are not properly coordinated. Considering this, the optimal MPR becomes particularly important to achieve the best system benefits, according to which transportation agencies could adopt appropriate policies to promote or inhibit CV development.

To meet the increasing technological requirements for mixed traffic, infrastructure-aided solutions using roadside equipment (RSE) are critical for enhancing CV performance and traffic efficiency. The RSE, equipped with sensing and communications devices, can sense vehicles along its covered road section and communicate the beyond-line-of-sight motion information to CVs. It can collect vehicle-based information, which is not limited to kinematic parameters but includes also the measurements stored by vehicles’ sensors, to estimate and monitor the traffic conditions in their coverage areas. It provides the basis for providing wide-ranging and high-resolution lane-level information. It can help promote CV deployment by providing vehicles with scalable communication, sensors, and computational support. It is also a traffic information collection method that is highly likely to be widely deployed in the future. With the development of intelligent transportation and smart roads, most roads have already been equipped with point-based roadside devices over years. However, the service life of this equipment is usually no more than 15 years [5,6]. With the development of CVs, this equipment may undergo several rounds of maintenance, replacement, and upgrades. In addition, as the proportion of CVs increases, the data provided by CVs can meet the information requirement. At that time, data missing caused by partially damaged roadside equipment might no longer be a major issue affecting data quality. Hereupon, to explore this issue, it is necessary to analyze the relationship among the MPR of CVs, the failure rate (FR) of roadside equipment, and data quality.

Although scholars such as Alemazkoor et al. [7], Salari et al. [8], Ding et al. [9], and Zou et al. [10] have put forward relevant sensor failure viewpoints, in general, they did not take the MPR of CVs into account. Some scholars discussed the impact of CV MPR on traffic delay [11,12,13], traffic safety [1,14], and the capacity improvement [15,16], but overlooked the influence of roadside sensors or sensor failure. However, delving deeper into the mutual influence among the MPR of CVs, the failure rate of roadside equipment and data quality is of great significance for the development of CVs and the deployment of RSE. Few studies have suggested a causal relationship among these indicators or measured their contribution. Moreover, there are numerous studies addressing the issue of sensor location with failure consideration in road networks. Nevertheless, there has not been much research in this regard on road sections, especially on highway corridors. To fill this gap, this study proposed a simulation-based approach combined with several data processing algorithms to measure the impact of CV MPR and sensor failure on data accuracy. To optimize the locations of sensors on a freeway corridor, an optimal deployment method with spatial constraint is proposed and is solved by an improved Simulated Annealing Genetic Algorithm (SAGA). The main contributions of this work are as follows:

(1) This is the first study to weigh the impact of CV MPR and the failure rate of RSE on data accuracy for the mixed traffic flow with CVs and human-driven vehicles on a freeway corridor.
(2) A general optimization model, which refers to the spatial uniformity and error minimization as objective and subject to spacing constraints, is formulated to find the optimal sensor deployment scheme. It can better balance the spatial selection fairness of sensor locations by subdividing road segments into cells with relatively small spacing. By solving the model, an improved SAGA is given to find the global optimal solution. This method is more suitable to address the equipment optimization issue at the road segment level.
(3) According to whether missing values are allowed or not, a rigid nearest neighbor algorithm and a soft nearest neighbor algorithm are proposed to handle the missing data caused by sensor failure. Based on these methods, the issue of missing single-source data at the road segment level can be addressed. Compared with missing RSE data imputation, the fusion with CV data can better enhance data quality.
(4) The key parameters—CV MPR and sensor failure rate—affecting data accuracy are discussed in detail. The findings shed light on the trade-offs and benefits associated with improving RSE deployment and promoting CV development.

The remainder of this paper is organized as follows. Section 2 provides an overview of the related works. Section 3 elaborates the methodology framework and relevant data-processing approaches. Section 4 conducts the experiments and investigates the impact of CV MPR and RSE failure rate on data accuracy. Finally, Section 5 concludes the paper.

## 2. Literature References

### 2.1. RSE Allocation Methods

The deployment of RSE has been studied for years with a view to traffic flow observability [17,18], travel time estimation [19,20,21], network coverage [18,22,23], and origin–destination estimation [24] and so on. The commonly used deployment methods in the early stages are the fixed-spacing method and the hotspot method. With the change in demand and the development of technology, optimizing sensor deployment is essential to use as few devices as possible while meeting the requirements under budget constraints. Various models and algorithms, the dynamic programming model [25], the stochastic model [24], the integer programming model [19,26,27], the heuristic algorithm, the genetic algorithm [28] and the simulated annealing algorithm [29], and the clustering algorithm [30], were proposed to solve the sensor allocation problem. Recently, the cooperative vehicle-infrastructure system has put forward new requirements for the deployment of roadside equipment. RSE plays a crucial role in enabling vehicle-to-infrastructure communication, as it can gather and broadcast traffic information in the road network. Methods for the deployment of roadside equipment are proposed in terms of road coverage [31,32,33], vehicle connectivity [29,34,35], and V2I communication performance [36]. The mathematical programming models [37,38] and heuristic algorithms [39,40] are still the popular methods.

### 2.2. The Influence of CV MPR on Traffic Application

In terms of CV MPR, one commonly discussed benefit is to improve traffic operations (that is, improved efficiency, decreased congestion, and delay) with the varying MPR of CVs in the mixed-flow traffic conditions. Stern et al. [41] studied the effect of AV (Automated Vehicle) MPR on traffic control and found that 5% or less of the AVs could control the traffic flow. Zheng et al. [42] analyzed the impact of AV MPR on uncertainty and instability of mixed traffic. They concluded that higher MPR can reduce the uncertainty inherent in human-driven vehicle behavior and improve the stability of mixed traffic flow. Argote-Cabanero et al. [11] proposed a method to determine the minimum penetration rate of CVs to estimate traffic speed and traffic delay accurately, and found that 15% would meet the target. Stanek et al. [12] found that 30–50% of AVs yield a decrease in network delay and an increase in network speed, which is almost the same as the 100% AV case. Abdeen et al. [13] concluded that vehicle delays were reduced by 26%, 34.4%, 63.7%, and 74.2%, when the MPRs of AVs are 25%, 50%, 75%, and 100%. Gao et al. [43] studied the real-time queue length at signalized intersections by taking connected vehicles as the mobile sensor, and found that the sensing accuracy is proportional to the penetration rate. Similarly, Željko Majstorović et al. [44] concluded that the accuracy of the speed transition matrices could be increased by the CVs penetration rate, but not linearly. Di et al. [45] used the average total travel time on the mainline and the average queue length of on-ramps as the indicator to optimize CV MPR, and found that the optimal MPR tends to be high when the weight of total travel time is high. Yao et al. [46] analyzed the stability of the mixed traffic flow with different automation levels, and found that the mixed traffic flow is more unstable than traditional human driving traffic flow when the CAV MPR is 80%. However, with the increase in MPR and the automation level of CAVs, mixed traffic flow tends rapidly to a stable state.

Some studies pay more attention on the influence of the MPR of CVs on traffic safety. Ye et al. [14] evaluated the impact of the connected and automated vehicle (CAV) penetration rate on traffic safety and found velocity difference between vehicles is decreased and traffic flow is greatly smoothed with the increase in the CAV MPR. Mousavi et al. [1] found that the number of rear-end conflicts and lane-changing conflicts were significantly decreased with the increase in the AV MPR. Chin et al. [47] found that less than 40% of CV MPR provided a safer traffic network in special conditions to maintain the standard mobility. Xiao et al. [48] proposed a meta-analysis approach to estimate the safety effects of intelligent CVs by MPR, and found the number of conflicts is exponentially reduced with the increase in the MPR. They expect the proportion of CVs in 2025 and 2035 will be 17–20% and 40–48%. Zhang et al. [49] found that adopting AVs at a low MPR of 2% can reduce 90% of the parking demand for clients. Yang et al. [50] observed that the likelihood of secondary crashes can decrease by up to 33% under high-volume conditions, when the MPR hits 25%. Papadoulis et al. [51] evaluated the safety impact of CAVs by developing a decision-making CAV control algorithm in the simulation software VISSIM, and found that estimated traffic conflicts were reduced by 12–47%, 50–80%, 82–92%, and 90–94% for 25%, 50%, 75%, and 100% CAV penetration rates, respectively.

There are also some other works researching the capacity improvements under different penetration rates of CVs. Jiang et al. [15] proposed a cellular automata model to study the influence of CAVs on mixed traffic flow. They found that the road capacity under the pure CAV environment has increased by 3.24 times compared with the pure HDVs, and the velocity fluctuation decreases significantly when the MPR reaches 80%. Liu et al. [16] concluded that the road capacity did not improve much when the penetration rate is less than 60%, but it increases by 90% with 100% CV. Shladover et al. [52] pointed out that the capacity improvements can result from different CAV MPRs due to potential vehicle platooning and reduction in the space required for CAVs on the road network. They found that the capacity can be improved by 22%, 50%, and 80% with the MPR of 50%, 80%, and 100%, respectively.

### 2.3. Sensor Failure on Traffic Data Acquisition

Sensor failure, as the main factor affecting traffic data perception, has been considered for several years. Most of the studies addressing intermittent equipment failures preferred to give the methods for missing traffic data imputation [23,53,54,55] and sensor allocation on a network [18]. Li et al. [56] considered the sensor failure probabilities in selecting sensor locations on a network for OD flow surveillance. They found that the path coverage was sensitive to sensor failure and installation budget, and high sensor failure probabilities showed a tendency to cluster sensors. Zhu et al. [57] proposed a stochastic conditional value at risk model to optimize the sensor locations on a freeway corridor. They found that considering sensor failure improves the placement pattern. Alemazkoor et al. [7] proposed an online recursive regression approach to predict the traffic flow for locations with faulty sensors by using neighboring sensor data solely, and found the prediction accuracy can generally reach 95%. An et al. [23] introduced a mixed integer linear programming approach to improve data accuracy by considering the risk of sensor failure. By minimizing the effect of sensor failure on the link flow inference of unobserved links, Salari et al. [18] proposed a method to identify the minimum set of links in a traffic network to reach full link flow observability. They suggested that minimizing the number of sensors for unobserved link and installing advanced sensors on links involved in the link flow inference is a solution for full link flow observability. On this basis, Salari et al. [8] applied a non-homogeneous Poisson distribution to deal with the time-varying failure rates of count sensors, and established a model to identify the optimal sensor locations for OD demand estimation. They found that the budget and the locations of damaged sensors play a key role in repairing failed sensors or installing new sensors. Sun et al. [58] proposed new robust models for deploying multi-type sensors by considering the factors such as sensor failure rate, sensor type, OD demands, and budget constraints. The Weibull distribution was used to characterize the time-varying sensor failure rate, and a nonlinear least squares method was utilized to estimate the parameters of the failure rate function. However, their research mainly focuses on the occasional sensor failure instead of physical damage. Ding et al. [9] gave a deep-learning approach to identify and eliminate redundant sensors in a traffic network, leading to a potential reduction in sensor nodes by 43% and connectivity edges by 82%. This implies that redundant sensor failure might not necessarily lower the benefits of the traffic networks. Zou et al. [10] proposed a framework based on long short-term memory and the multilayer perceptron to predict network-level traffic volumes with sensor failure. The results showed that the increase in mean absolute error (MAE) is less than 1 veh/5 min when the failure rate of the detector increases to 20%. Liang et al. [59] considered the possibility of roadside unit failures when deploying the roadside unit in a road network for traffic event information transmission. They found that decentralized deployment is suitable for low failure probabilities, while centralized deployment is preferred for high failure rates.

## 3. Methodology

### 3.1. The Optimal Deployment Method

Data accuracy and completeness, as exclusive factors for deploying sensors, directly affect the decision control system’s assessment of traffic conditions. The most commonly used parameter in evaluating data accuracy is travel time in consideration of its significance in determining traffic parameters and traffic state estimation. In terms of RSE deployment, a wealth of literature has provided various methods and measures. The uniform method, also called the fixed length method, is straightforward. A uniformly fixed spacing, for example 1000 m, was chosen as the unit length. However, this method may result in biased traffic condition estimations [60]. The hotspot deployment method, in which RSE is positioned at the accident-prone areas, the on-/off-ramps, or the crowded areas, is largely used due to budget constraint. The biggest benefit of this method is that it is inexpensive to deploy and maintain. Nevertheless, it is challenging to provide precise data for vehicle and road control. Another commonly used approach is the optimal method. It is used to find an optimal solution to an objective function subject to given constraints. Different optimization methods have their own advantages and are suitable for different scenarios and problem types. Based on this method, the locations of a finite set of sensors are optimized by minimizing the estimation errors of traffic parameters. Assuming the road is divided into cells with equal length, and each cell is considered as a candidate location for sensor deployment. The cells are numbered from 1 along the direction of traffic flow, facilitating the confirmation of the specific locations of roadside equipment. The selected deployment locations of RSE serve as the dividing points for sub-segments. The objective function in this study is to minimize both the expectation and the variance of the mean absolute percentage error (MAPE) of travel time. Not only does this function reduce the overall error, but it also lowers the error for each individual section. It should be noted that during the optimization process, adjacent cells may be selected as deployment locations. If the cell length is too small (such as 50 m or 100 m), the deployment plan with adjacent cells is meaningless. However, if the length is too large, some location points have no chance to enter the optimization process. This being thought of, a constraint (8) is adopted to ensure the minimum spacing of sensors no less than the communication range, *r*, to fully utilize the limited roadside equipment. In this case, even if the cell length is relatively small, there will be no excessively small positional spacing. In addition, to achieve spatial uniformity, spacing variance, Var(l), has been added to the objective function. The optimization model is as follows:

(1) Minimize F(N)=a∗(1n∑i=1nMAPEi)+b∗Var(MAPE)+c∗Var(l)

(2) $$\mathrm{where}\;{MAPE}_{i}=\frac{1}{m}\sum_{j=1}^{m}\frac{\left|T_{ji}-T_{i}\right|}{T_{i}}$$

(3) $$Var(MAPE)=E{\left[(MAPE-\frac{1}{n}\sum_{i=1}^{n}{MAPE}_{i}\right)}^{2}]$$

(4) $$Var\left(l\right)=\frac{1}{N-1}\sum_{i=1}^{N-1}{\left(\frac{l_{i,i+1}}{L}-\frac{1}{N-1}\sum_{i=1}^{N-1}\frac{l_{i,i+1}}{L}\right)}^{2}$$

(5) $$\mathrm{Subject}\mathrm{to}a=10^{-\left[lgE(MAPE)\right]-1}$$

(6) $$b=10^{-\left[lgVar(MAPE)\right]-1}$$

(7) $$c=10^{-\left[lgVar(\frac{l}{L})\right]-1}$$

(8) $$2r\leq l_{i,i+1}$$

(9) N≤n+1

(10) m,n∈N+

In the above formulas, *n* refers to the link number related to the number of sensors; *m* is the time series number, which is equal to the total sampling time divided by the sampling interval; *a*, *b*, and *c* are real numbers that keep the expectation and the variance of MAPE, the variance of adjacent sensor spacing, *l*, at the same order of magnitude; *N* is the number of RSE subject to budget constraints; $l_{i,i+1}$ is the spacing between sensor *i* and sensor *i* + 1; *L* is the length of the road. Equation (2) is the mean absolute percentage error on link *i*; Equation (3) is the variance of MAPE for all sections of the entire road; Equation (4) is the variance of sensor spacing. It is used to prevent the equipment locations from being too concentrated or too far apart.

Heuristic optimization methods (such as the genetic algorithm (GA) and the simulated annealing (SA) algorithm) perform well in solving complex nonlinear and multi-objective optimization problems. They can find near-optimal solutions in a relatively short time through empirical rules and random search. GAs simulate the natural selection and genetic mechanisms in biological evolution, iteratively generating better solutions through selection, crossover, and mutation operations. They have strong parallel search capability, making them suitable for handling complex problems. However, they are prone to premature convergence in immature regions, leading to local optima. SA is a probabilistic technique used to avoid becoming stuck in local optima during the search process. At high temperatures, SA allows for a greater probability of accepting inferior solutions, avoiding becoming stuck in local minima. As the temperature decreases, the probability of accepting inferior solutions also decreases. It becomes more focused on fine-tuning the current solutions. SA has low dependence on initial solutions and can escape from local optima during the search process. However, it is less efficient in high-dimensional search spaces. Combining the global search capability of SA with the parallel search and diversity maintenance capabilities of GA, the simulated annealing genetic algorithm (SAGA) might be an appropriate method for solving the optimization model.

**Figure 1:**
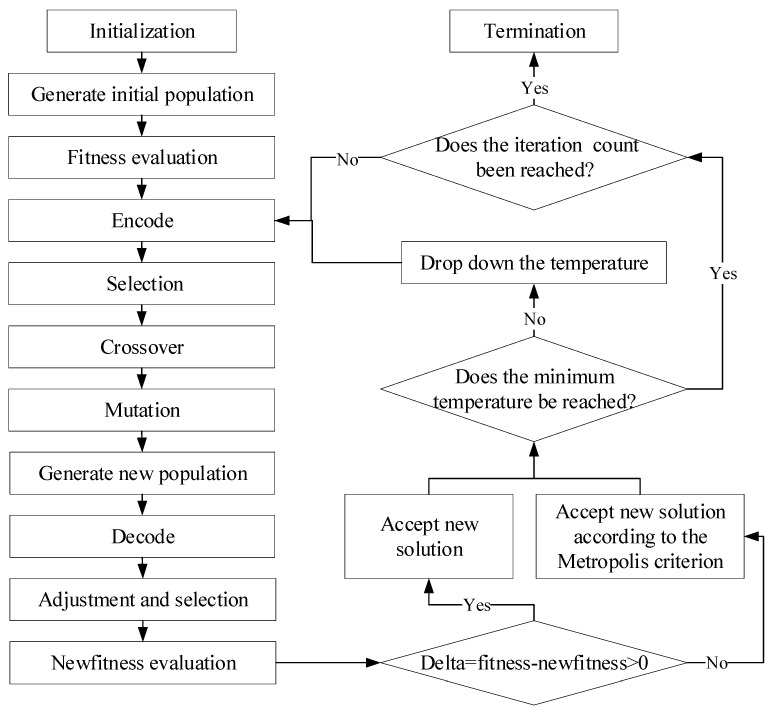
The SAGA flowchart.

The flowchart of SAGA is shown in Figure 1. The left half represents the process of GA, while the right half represents the process of SA algorithm. First, the initial parameters, including the population size, the number of sensors, crossover rate, mutation rate, temperature and its attenuation coefficient, and the number of iterations, and so forth, are all given. The next step is to randomly generate a set of solutions in the solution space as the initial population. One thing to note here is that individuals in the population should meet constraint (8). If not, adjustments need to be made. For example, if the spacing between sensor *i* and sensor (*i* + 1) is less than 2*r*, the position of sensor (*i* + 1) is adjusted so that the spacing is equal to 2*r*. In this case, if the distance between the location of sensor (*i* + 1) and the end of the road is less than 2*r*, the position of sensor *i* is adjusted instead of sensor (*i* + 1). The fitness of the algorithm is the objective function, F(*N*). The following steps—encoding, selection, crossover, mutation, and decoding—are the normal process of GA. Before calculating the fitness value of the new population, selection and adjustment should also be adopted to make sure the individuals with aberrant values be excluded from the population. After each iteration of the genetic algorithm, the acceptance criteria of SA are applied to the individuals in the current population to escape from local optima. Based on current temperature and fitness value, the new solutions are accepted if the new fitness values are smaller. Otherwise, the inferior solutions are accepted with a certain probability according to the Metropolis criteria. The algorithm terminates when the number of iterations is met.

### 3.2. The Simulation-Based Approach Combined with Data Processing Algorithms

Sensor failure involves two cases: the sensor is damaged and the sensor occasionally fails [61,62]. In the former case, the sensor cannot function properly, and no traffic data will be collected. In contrast, a small number of traffic data will occasionally be missing in the latter case. The lifespan of a sensor is usually represented by the Bathtub curve and can be conceptually divided into three stages: the initial damage stage, the effective lifespan stage, and the decay stage [63]. During the initial stage, also called the running-in period, the failure rate experiences a rapid decrease after continuous trial operation. Then, the failures occur randomly without predictable timing but tend to converge towards a constant value after being observed over a period. Finally, the failure rate increases rapidly to a very high value since the sensor has reached the stage of aging and wear. This study primarily focuses on the damage failure of sensors in the second to third stage.

Assume the failure rate of roadside equipment is $FR\left(t\right)$, which is a random number that increases over time. For the road with previously existing sensors, the total number of RSE on the road is N(0), and the number will be $N\left(t\right)=\left|N\left(0\right)(1-FR(t))\right|$ after a period of time (where || indicates rounding down). The data accuracy is influenced by the parameters, such as the number of RSE, the location of RSE, sensor type, and the MPR of CVs. Hereupon, the relationship among these indicators can be expressed as follows:

(11) $$A\left(t\right)=f\left(N\left(t\right),\;L\left(t\right),\;P_{CV}\left(t\right)\right)+\varepsilon$$

In the formula, A(t) is the overall data accuracy of a transportation network at time t. L(t) is the location matrix of RSE at time *t*. It is obtained by eliminating the positions of damaged equipment, $L_{d}(t)$, from the initial positions, $L\left(0\right)$. $P_{CV}(t)$ is the MPR of CVs at time *t*. ε is a stochastic disturbance.

**Figure 2:**
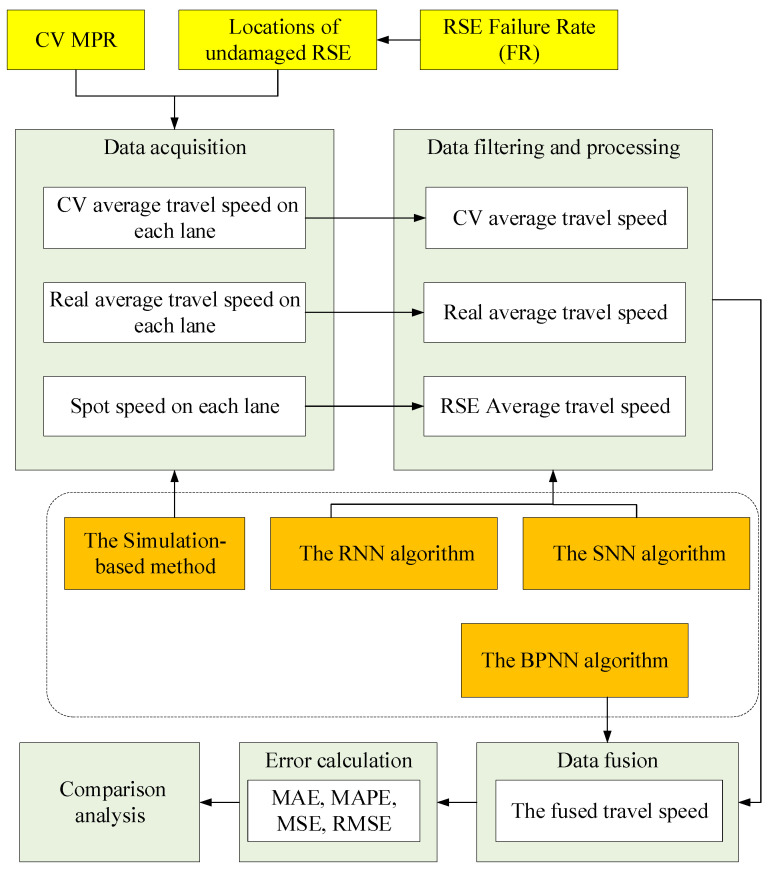
The approach framework.

To observe their relationship more intuitively, this study proposed a simulation-based approach combined with several data-processing algorithms to address the data performance under different CV MPRs and different sensor failure rates. The framework of the approach is shown in Figure 2, and the specific steps are as follows:

Step 1. Data acquisition. Based on the road geometry, traffic parameters of the target road, including traffic volume, driving speed, and vehicle types, as well as existing sensor information, are used to establish a simulated environment. The spot speed data and the road segment data at different CV MPRs can be obtained with the help of the simulation tool.

Step 2. Data filtering and data processing. Given that this research mainly focuses on the effect of travel time and travel speed, the spot speed collected by RSE and the average travel speed provided by CV and all types of vehicles are screened from the simulation data. Since the data is collected in separate lanes, the spot speed can be obtained by using Equation (12), which is a weighted arithmetic average value of the instantaneous speed of vehicles passing a point of the road over some specified time. Similarly, the average speed of all vehicles occupying a given section of the road, also called the travel speed, can be calculated by Equation (13).

(12) $${\bar{v}}_{p}=\frac{\sum_{j=1}^{n}q_{j}v_{j}}{\sum_{j=1}^{n}q_{j}}$$

(13) $${\bar{v}}_{s}=\frac{\sum_{j=1}^{n}{q_{j}^{\prime }v}_{j}^{\prime }}{\sum_{j=1}^{n}q_{j}^{\prime }}$$

In the formulas, *n* is the lane number. $q_{j}$ is the number of vehicles passing a point of lane *j*. $v_{j}$ is the instantaneous speed of vehicles passing a point of lane *j*. $v_{j}^{\prime }$ is the travel speed of vehicles on lane *j*. $q_{j}^{\prime }$ is the number of vehicles passing through a section of lane *j*. To conduct a comparative analysis, it is necessary to convert the point speed into the travel speed. Equation (14) is a solution to estimate the travel speed by using the spot speed. However, it is only applicable for calculating non-zero values. Given that there will be a significant amount of data missing in this study, Equation (15) is adopted to estimate the travel speed.

(14) $${\bar{v}}_{i,i+1}=\frac{2}{\frac{1}{v_{i}}+\frac{1}{v_{i+1}}}$$

(15) $${\bar{v}}_{i,i+1}=\left\{\begin{array}{l} 0,{\;v}_{i}=0,v_{i+1}=0\\ \frac{2}{\frac{1}{v_{i}}+\frac{1}{v_{i+1}}},v_{i}\neq 0,v_{i+1}\neq 0\\ v_{i},{\;v}_{i}\neq 0,v_{i+1}=0\;\\ v_{i+1},{\;v}_{i}=0,v_{i+1}\neq 0 \end{array}\right.$$

**Figure 3:**
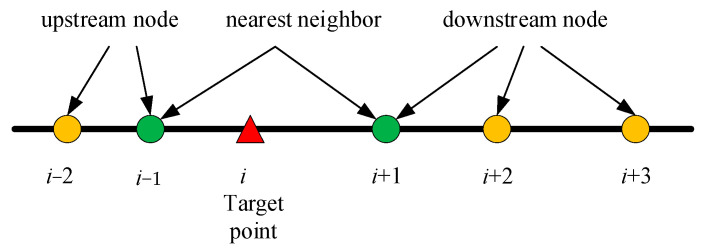
The nearest neighbors of the target point.

When it comes to dealing with the data-missing issue caused by sensor failure, the nearest neighbor (NN) algorithm is addressed. The nearest neighbor concept, particularly the KNN algorithm, is a fundamental technique in machine learning and data mining. Since the target road of this study is a freeway corridor, the distribution of data collection points exhibits a strong upstream-downstream relationship. In addition, this study assumes that no data will be collected from the sensor once it is damaged. Moreover, the data volume is not particularly large. Taking these factors into consideration, the nearest neighbors algorithm is more suitable for solving the problem of data missing in this situation. In this study, two points, namely the upstream node and the downstream node of the target point, were involved. As shown in Figure 3, the red triangle is the target point, and the two green circles are the nearest neighbor nodes of the target. According to the rule whether missing value on node is allowed or not, two methods are given to deal with the data. One is the rigid nearest neighbor (RNN) algorithm, in which only the nearest adjacent node (the green circle) in the upstream or downstream of the target position can be selected as the nearest neighbor. In this case, the missing data of the target node can be substituted for the one collected from the upstream or the downstream node. If the data collected by the adjacent nodes are all missing, no available data can be provided. According to this rule, the data collected from the target point, *i*, over time, *t*, can be obtained by Equation (16). In the equation, $V_{i}\left(t\right)$ is the speed matrix of node *i*, and O is the 0-matrix of the same rank. For a more detailed description of the algorithm, see Algorithm 1.

(16) $$V_{i}\left(t\right)=\left\{\begin{array}{l} V_{i-1}\left(t\right),\;V_{i+1}\left(t\right)=O\\ O,\;V_{i-1}\left(t\right)=V_{i+1}\left(t\right)=O\;\\ V_{i+1}\left(t\right),\;V_{i-1}\left(t\right)=O \end{array}\right.$$

**Algorithm 1** The rigid nearest neighbor algorithm**Input**: spot speed matrix $V_{p}$, damaged RSE location matrix $L_{d}$ **Output**: travel speed matrix $V_{s}$
1: Initialize the spot speed matrix $V_{p}$ based on $L_{d}$;
2: The elements in every two rows of the matrix $V_{p}$ are added and multiplied, marked as $V_{add}$ and $V_{multiply}$;
3: Find 0 in $V_{add}$ and $V_{multiply}$, marked the locations as $L_{common}$;
4: Find 0 in $V_{multiply}$ but not in $V_{add}$, marked the locations as $L_{uncommon}$;
5: Set the values in $V_{multiply}$ that are zero to 1;
6: Compute the $V_{s}$ according to Equation (16);
7: Set the values $V_{s}(L_{common})$ = 0;
8: Replace the row elements of $V_{s}$ with that of $V_{p}$ if $V_{p}(L_{uncommon})\neq 0$. otherwise,
9: Replace the row elements of $V_{s}$ with that of the next row of $V_{p}$;
10: Return $V_{s}$;

The other method is the soft nearest neighbor (SNN) algorithm. In the SNN algorithm, all the nodes around the target area will be traversed in sequence to find the nearest upstream node and the nearest downstream node with non-missing values. The travel speed for all sections between these two nodes has the same value. Briefly, if the RSE on node *i* − 1, node *i* and node *i* + 1 is all damaged, while on node *i* − 2 and node *i* + 2 it is not, then the travel speed of link (*i* − 2, *i* − 1), (*i* − 1, *i*), (*i*, *i* + 1) and (*i* + 1, *i* + 2) is equivalent to that of link (*i* − 2, *i* + 2). For a more detailed description of the algorithm, see Algorithm 2. Different from RNN algorithm, the SNN algorithm results in no occurrence of null values, even if there are multiple consecutive sections with equipment failures.
**Algorithm 2** The soft nearest neighbor algorithm**Input**: spot speed matrix $V_{N\times t}^{p}$, damaged RSE location matrix $L_{d}$ **Output**: travel speed matrix $V_{N-1\times t}^{s}$
1: Initialize the spot speed matrix $V_{N\times t}^{p}$ = {$V_{1}^{p};\;V_{2}^{p};...;V_{N}^{p}$} based on $L_{d}$;
2: For $\left(V_{i}^{p}\neq 0\;and\;V_{i+1}^{p}\neq 0\right)$, Compute the travel speed ${\bar{V}}_{i,i+1}$ according to Equation (15);
3: $for\;(V_{i}^{p}=0)$ do
4: Check if $V_{i-1}^{p}=0$ then
5: Repeat check until $V_{i-j}^{p}\neq 0$;
6: Check if $V_{i+1}^{p}=0$ then
7: Repeat check until $V_{i+k}^{p}\neq 0$;
8: Compute the travel speed ${\bar{V}}_{i-j,i+k}$ of link (*i* − *j*, *i* + *k*) according to Equation (15);
9: Assign the speed to all the sections between point (*i* − *j*) and point (*i* + *k*);
10: end for
11: repeat
12: Update the travel speed matrix $V_{N-1\times t}^{s}$;
13: until all elements in $L_{d}$ be considered
14: Return $V_{N-1\times t}^{s}$;

Step 3. Data fusion. Data fusion is a prevalent way to deal with imperfect raw data for capturing reliable and accurate information. Typical data fusion strategies, including the Bayesian estimation, Kalman filtering, Dempster–Shafer theory and some other machine-learning methods (such as neural networks), are often used to improve data quality. The neural network, which has excellent performance of powerful nonlinear fitting capabilities, self-organize, self-study, and strong generalization ability, is the most popular fusion method. Since images or complexly structured data are not involved, this study employs the Back Propagation Neural Network (BPNN) algorithm as the fusion algorithm. The steps are listed in Algorithm 3.
**Algorithm 3** The BPNN algorithm**Input**: travel speed from RSE, the CV travel speed, the true travel speed and the CV MPR **Output**: the fused travel speed
1: Splitting training data and testing data according to the train-test split ratio.
2: Normalization of training data.
3: Build a BP neural network with input layer, hidden layer and output layer. The transfer functions of the hidden layer and the output layer are tansig and purelin, respectively. The training is conducted by using the trainlm method.
4: Setting network parameters, such as the number of training iterations, learning rate, minimum error of training target, etc.
5: BP neural network training.
6: Normalization of testing data.
7: BP neural network prediction.
8: Prediction result normalization.

Step 4. Error calculation. To assess and measure the data quality, the indicators used in this study are MAE, MSE (Mean Squared Error), RMSE (Root Mean Square Error), and MAPE. The MAE measures the average absolute difference between predicted and actual values without considering their direction. it can accurately reflect the magnitude of the error. Compared with MAE, MSE squares each error before averaging which makes it more sensitive to large errors. RMSE is the square root of MSE. It puts the error back into the original units, which makes it easier to interpret than MSE. Like MSE, RMSE gives more weight to large errors. The MAPE is a variation of the MAE. MAPE not only considers the error between the predicted value and the actual value but also considers the ratio of the error to the actual value. This value is presented in the form of a percentage and is not affected by outliers. Since the dividing points of the road section are the locations of RSE, the amount of data for the predicted values is $N\left(t\right)-1$. The calculation formulas are Equations (17)–(20).

(17) $$MAE(t)=\frac{1}{N\left(t\right)-1}\sum_{i=1}^{N\left(t\right)-1}\left|{\hat{v}}_{i}-v_{i}\right|$$

(18) $$MSE(t)=\frac{1}{N\left(t\right)-1}\sum_{i=1}^{N\left(t\right)-1}{\left({\hat{v}}_{i}-v_{i}\right)}^{2}$$

(19) $$RMSE(t)=\sqrt{\frac{1}{N\left(t\right)-1}\sum_{i=1}^{N\left(t\right)-1}{\left({\hat{v}}_{i}-v_{i}\right)}^{2}}$$

(20) $$MAPE(t)=\frac{100\%}{N\left(t\right)-1}\sum_{i=1}^{N\left(t\right)-1}\frac{\left|{\hat{v}}_{i}-v_{i}\right|}{v_{i}}$$

Step 5. Randomly select the location(s) of the damaged equipment based on the failure rate and the number of RSE. To make the results robust and the data comparable between different experiments and research groups, the selection of the damaged position for the next round should retain the results of the previous round. In other words, the number of damaged sensors will increase by a fixed percentage during each round. Repeat steps 2–5 until all the pre-determined failure rates be considered.

Step 6. An error matrix, the rows of which correspond to the different sensor failure rates and the columns correspond to the different CV MPRs, will be addressed. The effect of CV MPR and the failure rate of roadside equipment on traffic perception accuracy can be obtained by analyzing the matrix data.

## 4. Case Analysis

### 4.1. Simulation Set-Up

**Figure 4:**
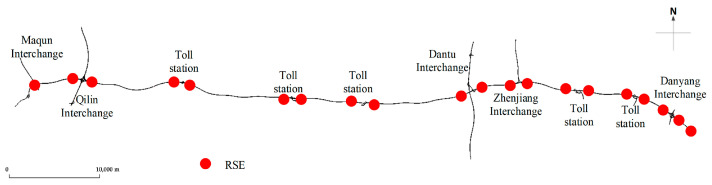
The sketch map of Nanjing–Danyang freeway with RSE deployed near the on-/off-ramps.

The software-based micro-simulation has been used to evaluate the feasibility and effectiveness of various approaches. The Simulation of Urban Mobility (SUMO), which is an open-source, highly portable, microscopic, and continuous traffic simulation package, is applied to construct relevant scenarios for obtaining required data. The simulation is conducted on a basic freeway segment from Nanjing to Danyang, which is a unidirectional four lane freeway with 74 km, in the mixed traffic environment including regular human-driven vehicles and CVs. For easy numbering, the road is divided into cells with equal length of 100 m, resulting in 740 road section numbers. The communication range of RSE is set as 250 m, which means the spacing between RSE is no less than 500 m. Figure 4 shows the sketch map of the simulated road. E1 detectors (induction loop detectors) are deployed on these cells to collect the information of vehicles passing the cross-section, and E2 detectors (lane area detectors) are used to collect the segmental level information. Additionally, different car-following models—CACC and IDM—are used for CVs and human-driven vehicles. The CVs, featured in the simulation, possess dimensions of 5 m in length and are configured with a maximum speed of 70 m/s, alongside maximum acceleration and deceleration rates set at 2.0 m/s^2^ and 2.0 m/s^2^, respectively. They adhere to a minimum distance of 2 m from the preceding vehicle. The human-driven vehicles, featured in the simulation, possess dimensions of 5 m in length and are configured with a maximum speed of 50 m/s, alongside maximum acceleration and deceleration rates set at 1.5 m/s^2^ and 1.5 m/s^2^, respectively. The lane-change model is LC2013. The MPR of CVs is from 10% to 100% with an interval of 10%. In addition, the values of 5% and 15% were also included to enrich low CV MPR scenarios. The detailed simulation parameters are shown in Table 1.

### 4.2. Failure Rate Setting and Data Processing

The failures of RSE occur randomly without predictable timing. However, after a period of observation, they tend to converge to a constant value. Instead of capturing the time-varying behavior of sensor failure, this work focuses on proving the impact of sensor failure on data accuracy under fixed failure rates. The failure rate of RSE ranges from 10% to 80% with an interval of 10%. To adhere to the principle of fairness, the damaged cell deploying with RSE is randomly generated based on the given failure rate. Assuming the damaged equipment will not be replaced or maintained during the period when CV MPR remains unchanged, the damaged cells selected from the previous round will be included in the next round. The randomly generated numeric sequence representing the failure sensors is {12, 9, 15, 19, 18, 5, 14, 1, 6, 4, 13, 8, 17, 7, 10, 20}.

As a highly functional and widely used tool, MATLAB R2022a is renowned for its powerful computing capabilities and features a distinctive matrix-oriented programming language. The library packages it comes with are much more extensive than those provided by other simple programming tools, and it also has a robust graphical user interface. These characteristics make it a better option for data processing and algorithm development. Therefore, data processing and data fusion in this study is achieved through programming in MATLAB R2022a. The data will be analyzed in detail in the next section.

### 4.3. Results Analysis

#### 4.3.1. Comparison and Analysis of RSE Deployment Methods

The locations of RSE obtained by using the uniform method, the hotspot method and the optimal method are listed in Table 2. In addition to the starting and ending positions, RSE is deployed near the ramps in the hotspot method (see Figure 4). The number of RSE locations is 20 and its location cells are listed in the third column of the Table. At this value, the spacing between two adjacent RSE placements is 3700 m which is selected as the parameter of the uniform method. The location of the cells can be seen in the second column. In the optimal method, the initial deployment scheme is randomly generated and is optimized by using the ASGA, the AS algorithm, and the GA. The initialization parameters of these optimal algorithms are listed in Table 3, and the results are listed in the last three columns of Table 2.

**Figure 5:**
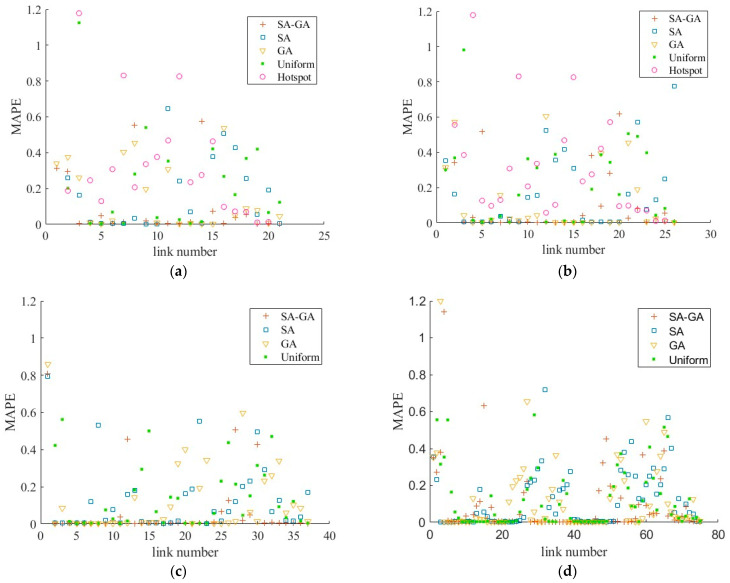
MAPEs of the data collected under different deployment schemes: (**a**) the number of sensors is 20; (**b**) the number of sensors is 25; (**c**) the number of sensors is 36; (**d**) the number of sensors is 74.

Figure 5 compares five sets of MAPEs for the data collected under different deployment schemes. In Figure 5a, the number of sensors is 20 and the locations of sensors are listed in Table 4. It can be seen from the figure that MAPEs of the SAGA are mostly distributed near the 0-axis. Compared with SAGA, the SA and the GA have higher MAPEs, almost half of which are distributed within the range of 0.2 to 0.4. Even though the MAPEs of the uniform method fall within the range of 0.2 to 0.4 as well, they have relatively higher values. The MAPEs of the hotspot method are nearly the highest on the first fourteen links, whereas on the last five links are the lowest except for the ones obtained by the SAGA. Therefore, we can draw a conclusion that the optimal method outperforms the uniform method and the hotspot method. To elaborate further, SAGA is superior to SA and GA. To verify the validity of the conclusion, the number of sensors has increased to 25 and the results are shown in Figure 5b. Five hotspots, whose cell numbers are {11, 86, 474, 640, 657} which correspond to the service area and the upstream and downstream areas of the Danyang bridge, are added to the initial hotspot scheme. Through comparison, it can be found that Figure 5b presents the same results as Figure 5a. Subsequently, the number of sensors is increased to 36, in which the average spacing between sensors is almost 2000 m, to observe the effect of the proposed optimization algorithms. For comparison, the uniform method is chosen since the locations are relatively dense, and the results are shown in Figure 5c. Similarly, the result is consistent with the previous conclusion. Figure 5d compares the MAPEs when the number of sensors is 74. Since the spacing between devices is already very small (e.g., 1000 m), the disparity in results between different methods was narrowed. However, it can still be observed that the errors of SAGA are smaller than those of other methods. As can also be seen from the figure, the error decreases as the number of sensors increases, which is consistent with previous research findings.

**Figure 6:**
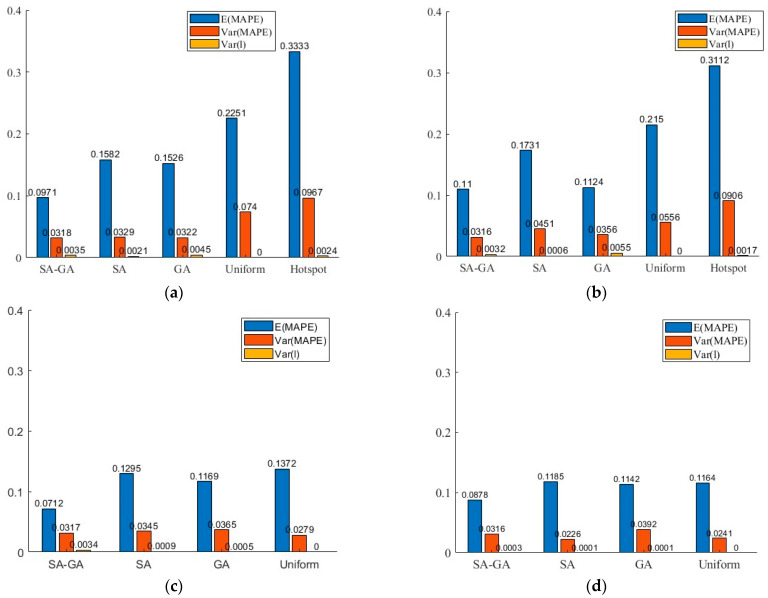
The expectation and variance of MAPE, and the variance of sensor spacing under different methods: (**a**) the number of sensors is 20; (**b**) the number of sensors is 25; (**c**) the number of sensors is 36; (**d**) the number of sensors is 74.

To further observe the performance of these schemes, Figure 6 gives the expectation and the variance of MAPE, and the variance of sensor spacing for each method. As shown in the figure, the SAGA undoubtedly performs the best, followed by the GA, the SA, the uniform method, and the hotspot method. On the whole, the optimal method outperforms the uniform method and the hotspot method, and the GA has an edge over the SA. Relatively speaking, the performance of SAGA is stable under different sensor numbers. The variances of MAPE and the sensor spacing of this method are basically unchanged. The expectation of MAPE fluctuates slightly, but it does not decrease continuously with increasing number of sensors. This is due to the need to strike a balance between spatial uniformity and data accuracy. Even so, it still performs the best among various methods. In addition, there is no doubt that the error will decrease as the number of sensors increases for the uniform and the hotspot method. By comparing Figure 6c,d, it is found that the differences in results between various methods have narrowed as the number of sensors increases. This is because a sufficient number will reduce the diversity of the samples, which in turn reduces the performance differences among different methods. In addition, although the number of sensors has doubled, the improvement in data accuracy is tiny. This indicates that simply increasing the number of sensors cannot continuously improve data accuracy. It is consistent with the findings of [21,60].

#### 4.3.2. The Impact of CV MPR on Data Accuracy

To observe the impact of CV MPR and equipment failure on data quality, this study chooses the deployment schemes obtained by the optimal method solved by the SAGA (called the SAGA deployment scheme in subsequent content) and the hotspot method as examples. The locations of RSE placements are listed in the third and the fourth column of Table 2. The RSE data and CV data at different MPRs are fused by using the proposed BPNN algorithm. The initialization parameters of this algorithm are listed in Table 4. The number of the input layer is three, corresponding to the three parameters of RSE data, CV data, and CV MPR. The output layer is the fused data. The number of the hidden layer is five, which is determined by using the trial-and-error method based on the numbers of the input layer and the output layer. For comparison, this study also adopted other machine-learning methods, such as the Long Short-Term Memory (LSTM) model and the Random Forest (RF) model, to fuse the data. In LSTM, the initial learning rate, the layer numbers, and the train-test split ratio are all the same as BPNN. In addition, the learning rate optimization algorithm is the adaptive moment estimation (Adam) algorithm, and the maximum epochs is 84 which is equal to the test population. In RF, the number of trees is set as 100 and the depth is 5.

**Figure 7:**
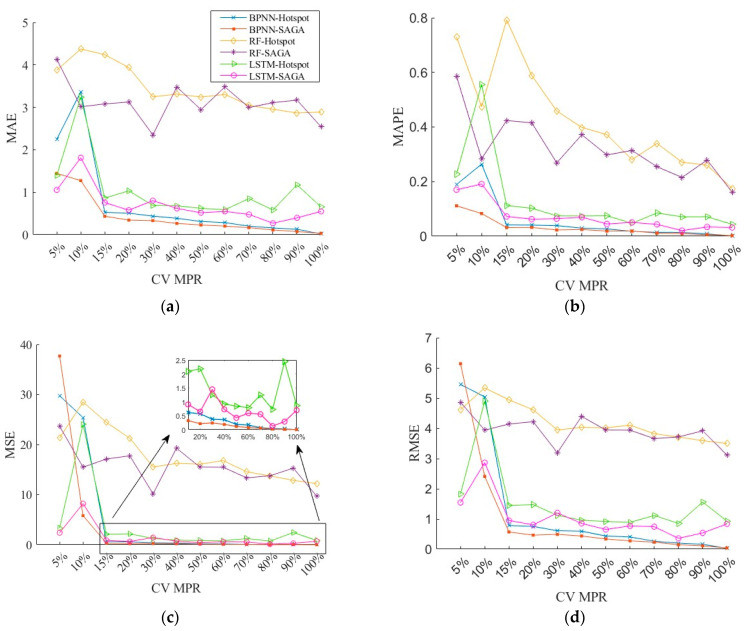
The results of different fusion methods: (**a**) MAE; (**b**) MAPE; (**c**) MSE; (**d**) RMSE.

Figure 7 plots four types of errors—MAE, MSE, RMSE, and MAPE—when the MPR of CVs varies from 5% to 100% for different methods. To enhance the robustness of the results, the mean value of the results obtained by running the program 10 times repeatedly for BPNN and LSTM is taken as the final value. For the RF, a random seed is added to the algorithm. It can be seen from the figure that the fused data obtained by using the BPNN algorithm substantially has the smallest errors, followed by the LSTM and the RF algorithm. When the MPR is lower than 10%, the LSTM performs better than the BPNN. Once the MPR exceeds 10%, the advantage of the BPNN algorithm becomes evident. It can also be clearly seen in the figure that the errors generally follow a downward trend with the increase in CV MPR. However, the fluctuations are large at different MPRs for RF in the SAGA deployment scheme, although the overall trend is downward. Therefore, this study chooses BPNN as the data fusion algorithm. To further observe the relationship between CV MPR and data accuracy, the results of BPNN are extracted separately from the figure.

**Figure 8:**
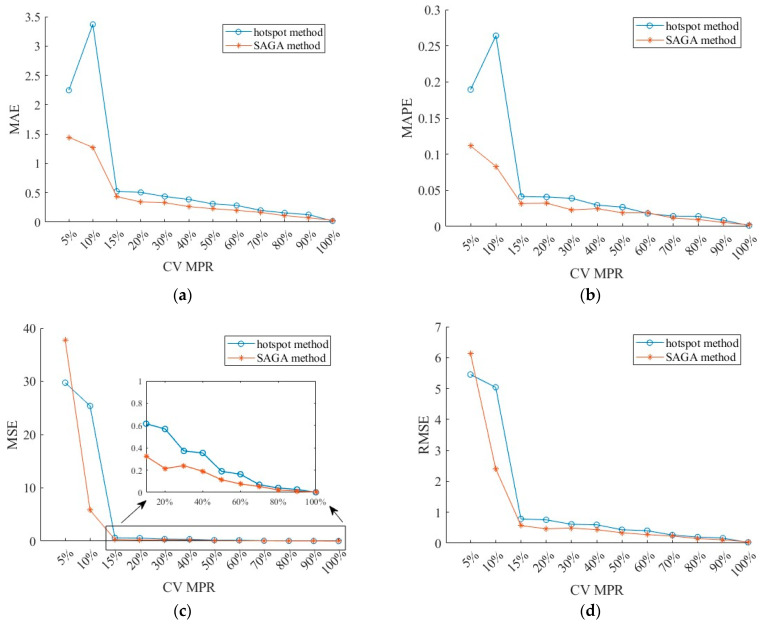
Errors at different CV MPR under the hotspot and the SAGA deployment schemes: (**a**) MAE; (**b**) MAPE; (**c**) MSE; (**d**) RMSE.

Figure 8 displays the MAE, MSE, RMSE, and MAPE of the fused data obtained by using the BPNN algorithm. It can be seen from the figure that the performance of the SAGA method is still superior to that of the hotspot method, and the errors generally follow a downward trend with the increase in CV MPR. However, when it exceeds 15%, the rate of decline is gradually slowing down. The MAPEs under the two deployment schemes are lower than 0.05 when the MPR is 15%, and they are lower than 0.02 when the MPR exceeds 60%. To put it another way, data accuracy can reach over 95% as the MPR is higher than 15%. Even though the number of CVs has increased from 15% to 60%, the accuracy has only improved by less than 2.5%. This implies that adding more CVs might not be able to significantly improve the data quality since the data accuracy is already quite high. Referring to RMSE, the value is lower than 1.0 as the MPR is higher than 15%, in which it is below 0.5 for the SAGA deployment scheme. When the MPR exceeds 60%, the RMSE is less than 0.5. After exceeding 60%, moreover, its rate of decline becomes even slower. At this point, we can safely draw this conclusion that 15% MPR is a turning point in improving data accuracy, which is in line with the research findings of [11], while 60% is another one.

**Figure 9:**
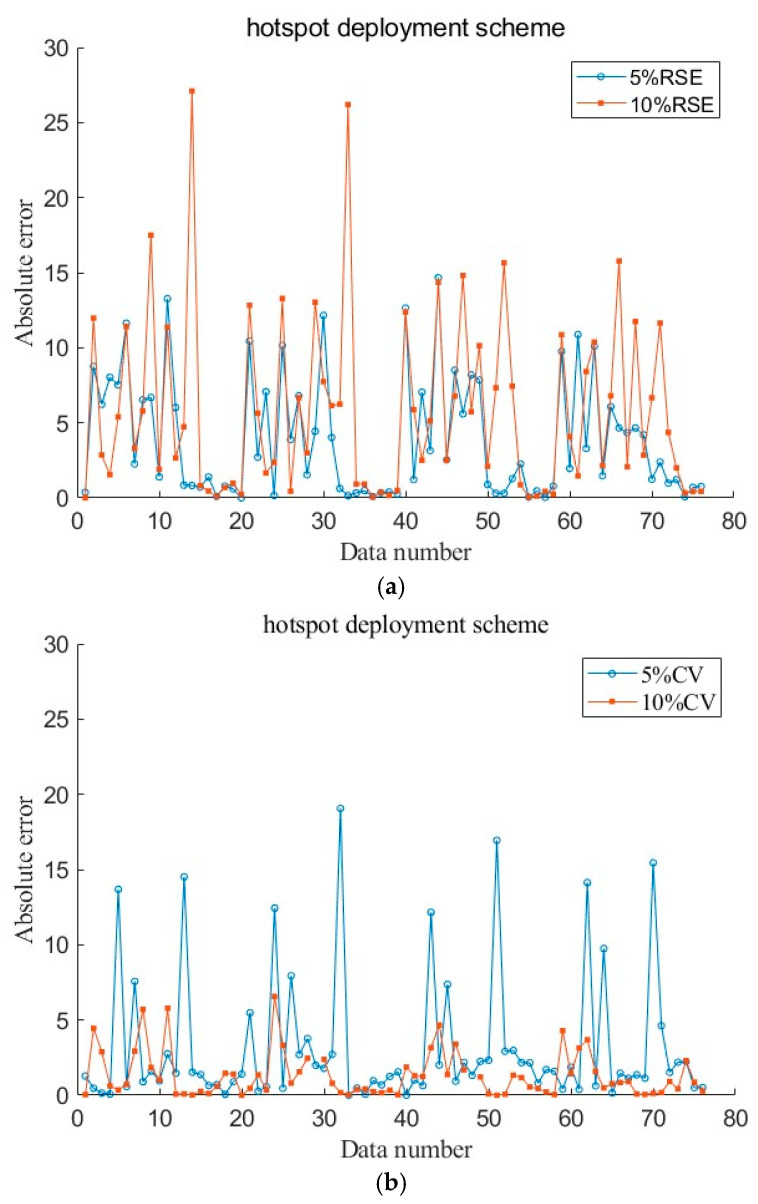
Absolute errors between raw data and test data at 5% and 10% CV MPR under the hotspot deployment scheme: (**a**) absolute errors between RSE data and test data; (**b**) absolute errors between CV data and test data.

As an unintended consequence, in addition, MAE and MAPE at 10% MPR are a little higher than that at 5% MPR under the hotspot deployment scheme. This discrepancy could be attributed to the interference of extreme errors on certain segments, which can be seen in Figure 9. Figure 9 shows the absolute error of the pre-fusion data collected by RSE and by CVs separately at 5% CV MPR and 10% CV MPR. The abscissa represents 76 data points from 19 road segments across four time periods. In the legend, 5%RSE refers to the RSE data and 5%CV refers to the CV data collected under a 5% CV MPR scenario. For convenience, we use the names in the legend to refer to each curve. In Figure 9a, 10%RSE data exhibit significant fluctuations and have a larger error than 5%RSE. However, the 5%CV data in Figure 9b exhibit obvious fluctuations and have a larger error than 10%CV. The high error of the source data pushes up the error of the fused data. Another point of anomaly is that the MSE and RMSE under the SAGA deployment scheme are a little higher than the hotspot scheme at 5% MPR. This is due to the MSE and the RMSE are sensitive to extreme values, only a few of which could elevate these errors.

#### 4.3.3. The Impact of RSE Failure Rate on Data Accuracy

**Figure 10:**
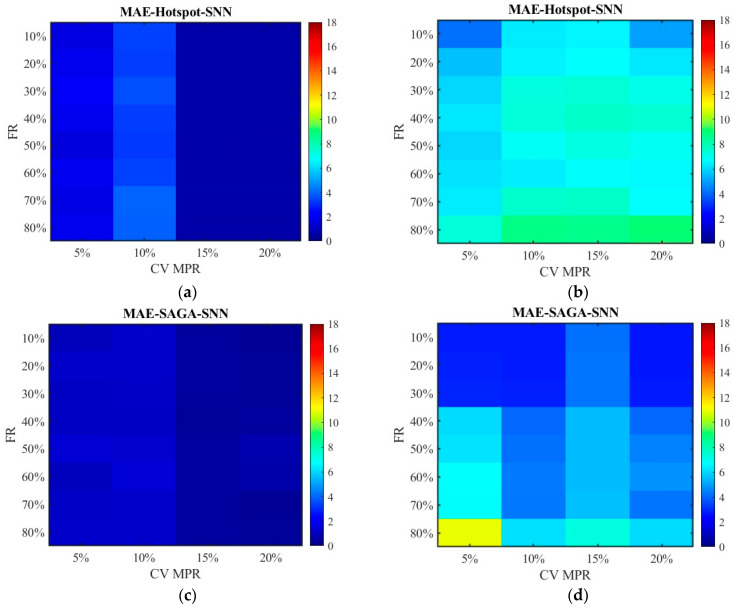
The distribution of MAE with the SNN algorithm: (**a**) MAE of the fusion data under the hotspot deployment scheme; (**b**) MAE of the pre-fusion data under the hotspot deployment scheme; (**c**) MAE of the fusion data under the SAGA deployment scheme; (**d**) MAE of the pre-fusion data under the SAGA deployment scheme.

**Figure 11:**
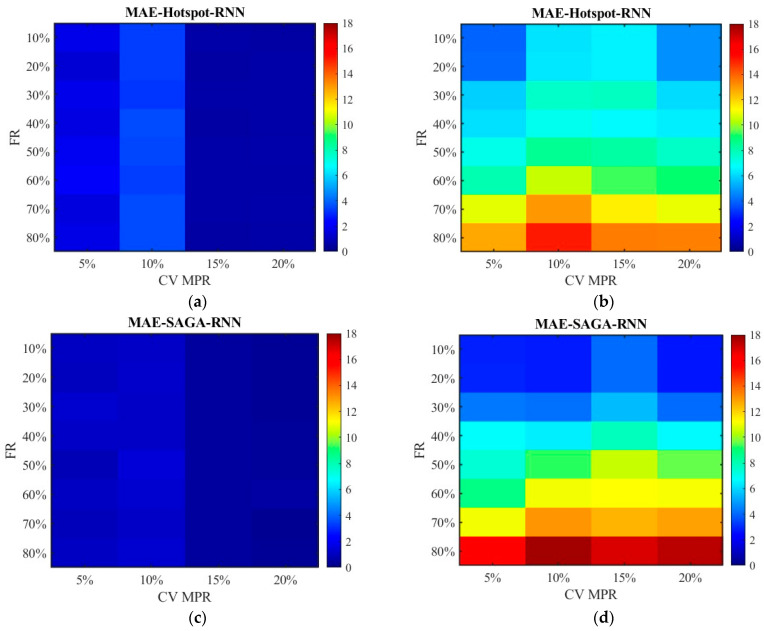
The distribution of MAE with the RNN algorithm: (**a**) MAE of the fusion data under the hotspot deployment scheme; (**b**) MAE of the pre-fusion data under the hotspot deployment scheme; (**c**) MAE of the fusion data under the SAGA deployment scheme; (**d**) MAE of the pre-fusion data under the SAGA deployment scheme.

**Figure 12:**
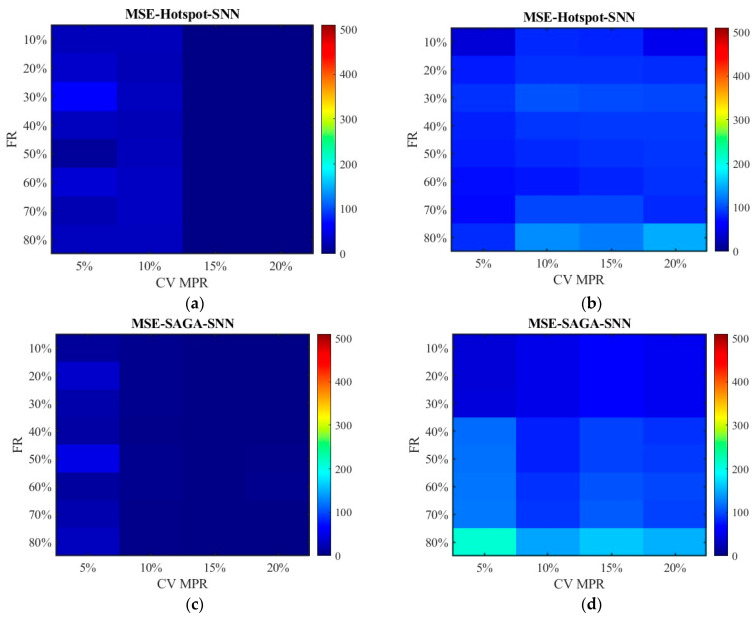
The distribution of MSE with the SNN algorithm: (**a**) MSE of the fusion data under the hotspot deployment scheme; (**b**) MSE of the pre-fusion data under the hotspot deployment scheme; (**c**) MSE of the fusion data under the SAGA deployment scheme; (**d**) MSE of the pre-fusion data under the SAGA deployment scheme.

**Figure 13:**
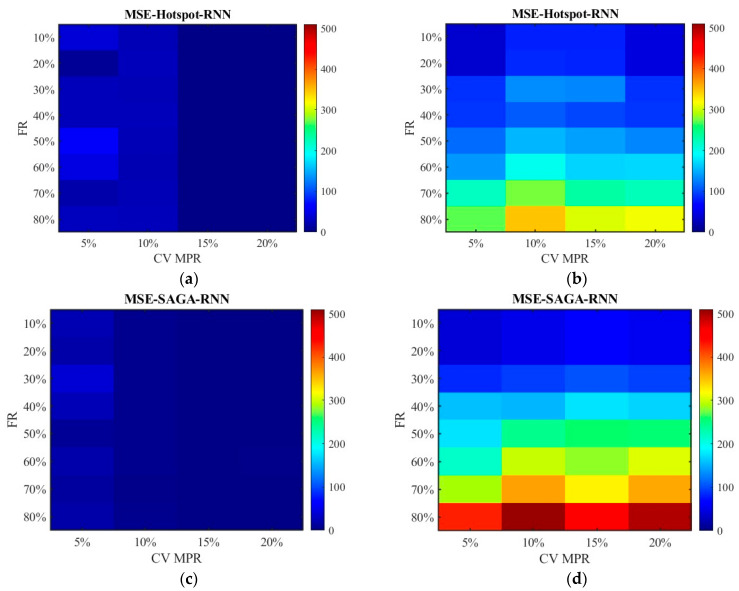
The distribution of MSE with the RNN algorithm: (**a**) MSE of the fusion data under the hotspot deployment scheme; (**b**) MSE of the pre-fusion data under the hotspot deployment scheme; (**c**) MSE of the fusion data under the SAGA deployment scheme; (**d**) MSE of the pre-fusion data under the SAGA deployment scheme.

**Figure 14:**
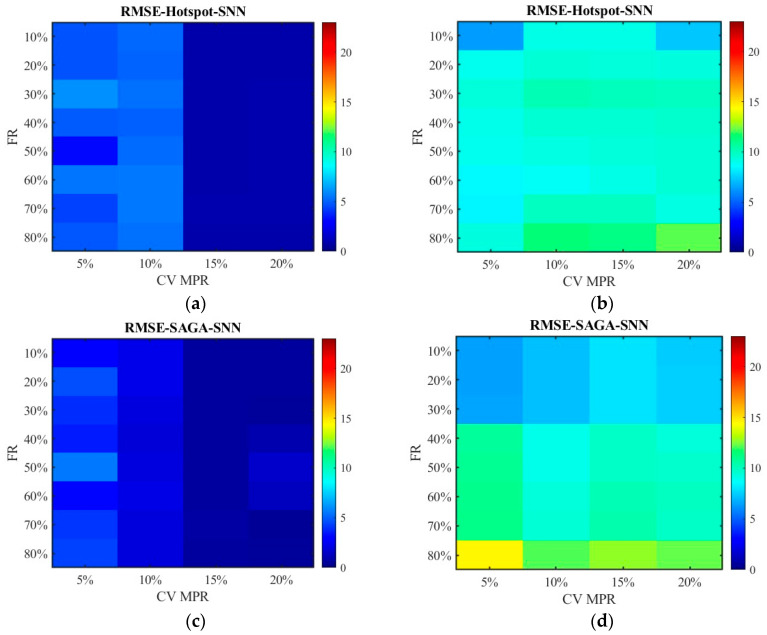
The distribution of RMSE with the SNN algorithm: (**a**) RMSE of the fusion data under the hotspot deployment scheme; (**b**) RMSE of the pre-fusion data under the hotspot deployment scheme; (**c**) RMSE of the fusion data under the SAGA deployment scheme; (**d**) RMSE of the pre-fusion data under the SAGA deployment scheme.

**Figure 15:**
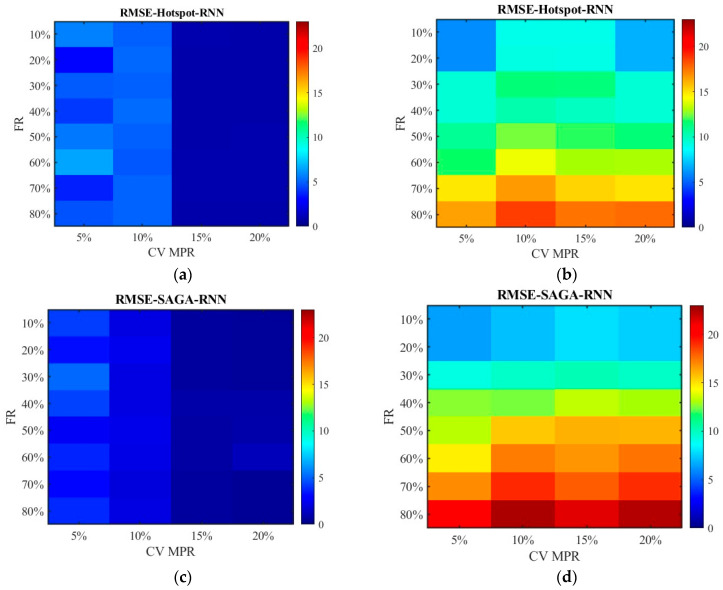
The distribution of RMSE with the RNN algorithm: (**a**) RMSE of the fusion data under the hotspot deployment scheme; (**b**) RMSE of the pre-fusion data under the hotspot deployment scheme; (**c**) RMSE of the fusion data under the SAGA deployment scheme; (**d**) RMSE of the pre-fusion data under the SAGA deployment scheme.

**Figure 16:**
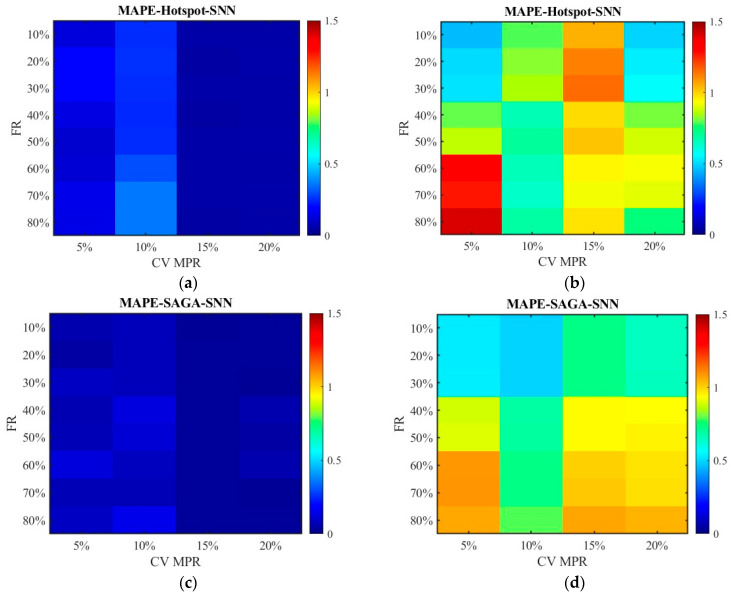
The distribution of MAPE with the SNN algorithm: (**a**) MAPE of the fusion data under the hotspot deployment scheme; (**b**) MAPE of the pre-fusion data under the hotspot deployment scheme; (**c**) MAPE of the fusion data under the SAGA deployment scheme; (**d**) MAPE of the pre-fusion data under the SAGA deployment scheme.

**Figure 17:**
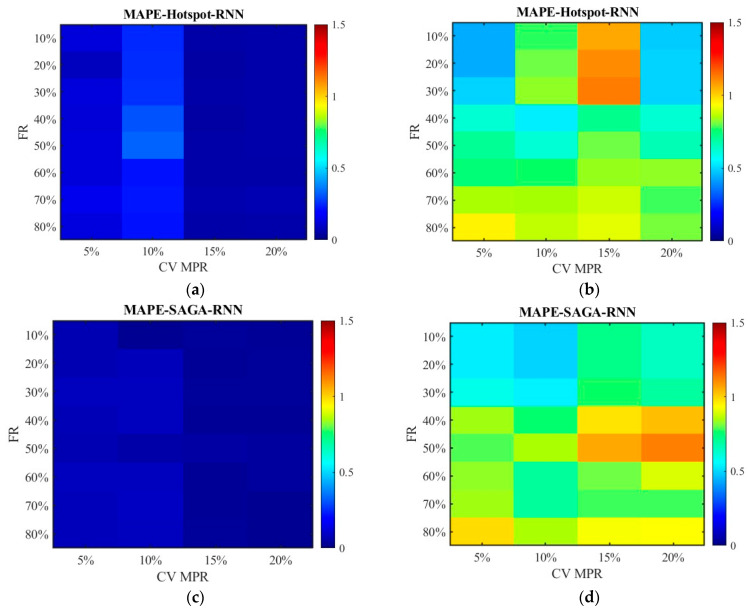
The distribution of MAPE with the RNN algorithm: (**a**) MAPE of the fusion data under the hotspot deployment scheme; (**b**) MAPE of the pre-fusion data under the hotspot deployment scheme; (**c**) MAPE of the fusion data under the SAGA deployment scheme; (**d**) MAPE of the pre-fusion data under the SAGA deployment scheme.

Based on the failure rates, the damaged locations of RSE given in Section 4.2 and the cells representing the deployment locations in Table 2, the spot speed data is filtered and fused with the CV data. The errors between the pre-fusion data, fusion data, and the true data at different RSE failure rates are calculated. From the above analysis, we already knew that data accuracy is relatively high when the CV MPR is above 15%. Thus, this section mainly focuses on the cases where the proportions are below 20%. Figure 10, Figure 11, Figure 12, Figure 13, Figure 14, Figure 15, Figure 16 and Figure 17 display the error distribution of MAE, MSE, RMSE and MAPE, among which Figure 10, Figure 12, Figure 14 and Figure 16 show the error distribution for the data processed by the SNN algorithm, while Figure 11, Figure 13, Figure 15 and Figure 17 show the data processed by the RNN algorithm. Each sub-graph in the figure represents the error of the data obtained under the hotspot deployment scheme and the SAGA deployment scheme. These data include both pre-fusion data and post-fusion data, where the left side shows the post-fusion data error and the right side shows the pre-fusion data error.

It can be seen from Figure 10 that the MAEs under the SAGA deployment scheme are lower than that under the hotspot deployment scheme regardless of whether the data is fused or not. Upon further comparison between fused data and non-fused data, it can be observed that the fused data exhibits smaller errors. For unfused data under the hotspot deployment scheme, the MAE increases as the failure rate increases. Although the fluctuation range of error values is small under the SAGA deployment scheme, it is also on a slow increasing trend. For the fused data, the MAE does not increase linearly with the increase in failure rate but rather experiences a slight fluctuating growth. When the proportion of CVs is 15% or higher, the MAEs remain relatively small and show little variation under different sensor failure rates. There are several possible explanations for this result. Data fusion is the most significant influencing factor, as it directly enhances the completeness, accuracy, and usability of the data by combining diverse data. Through data fusion, the missing data were supplemented by highly accurate connected vehicle data, which makes the accuracy of the data not greatly affected by the loss of data. Another possible explanation is that some missing data might be supplemented by their neighbors according to the SNN algorithm. Through this process, the data was not actually missing in a strict sense as long as the equipment around it is not damaged. There is also a possibility that the RSE data that have been excluded might correspond to the part with relatively large errors, or the damaged equipment is redundant.

It can be observed from Figure 10a,c and Figure 11a,c that the quality of data has been significantly improved with the increase in CV MPR. Only fusing 5% of the CV data has reduced the MAE of the RSE data from 2.85 to 15.65 in Figure 11d to 0.96–1.31 in Figure 11c, and from 3.98 to 12.74 in Figure 11b to 1.37–2.09 in Figure 11a. Although the error in 10% MPR is relatively large, due to the reasons mentioned in Section 4.3.2, it is still much lower than that of the unfused data. The MAE of the RSE data in 10% MPR is reduced from 10 to 3 on average. When the MPR reaches 15%, The MAE of the RSE data is reduced from 10 to below 0.5 on average. Based on the results, it can be inferred that multi-source data fusion, especially with the high-precision data, is much more effective in improving data accuracy than missing data imputation.

**Figure 18:**
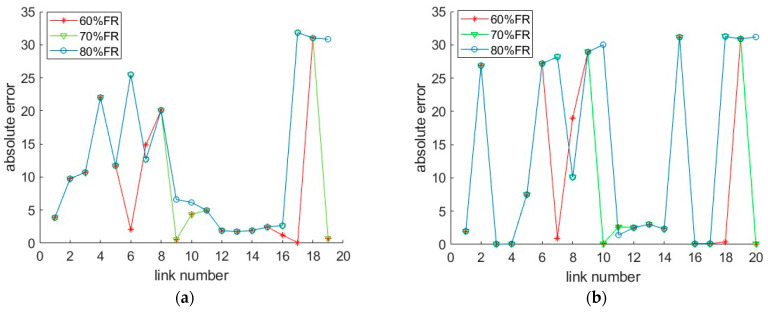
Absolute errors between the RSE data with 60–80% sensor failure rate and the true data at 5% CV MPR: (**a**) the hotspot deployment scheme; (**b**) the SAGA deployment scheme.

**Figure 19:**
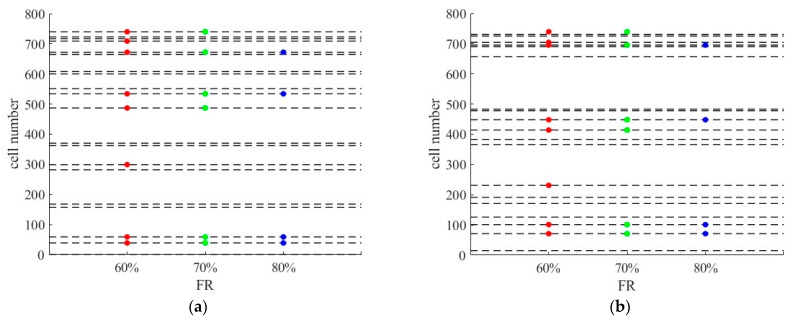
Locations of sensors at 60–80% sensor failure rate (the dashed line indicates the initial positions of sensors): (**a**) the hotspot deployment scheme; (**b**) the SAGA deployment scheme.

**Figure 20:**
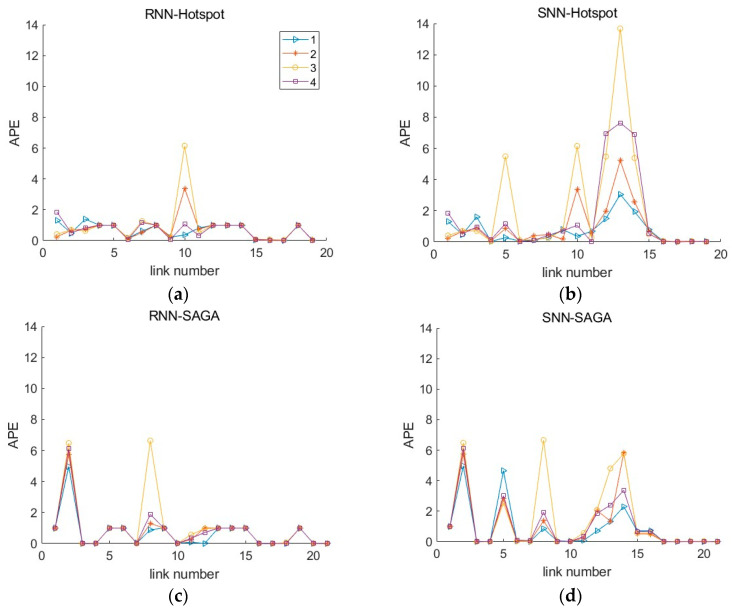
The distribution of APE (Absolute Percentage Error) of the pre-fusion data in 60% sensor failure rate when the CV MPR is 5%: (**a**) data processed by RNN under the hotspot deployment scheme; (**b**) data processed by SNN under the hotspot deployment scheme; (**c**) data processed by RNN under the SAGA deployment scheme; (**d**) data processed by SNN under the SAGA deployment scheme.

By comparing Figure 10b,d and Figure 11b,d, the data processed by the SNN algorithm is less sensitive to failure rate than the RNN algorithm. When the failure rate exceeds 40%, the error of the data processed by RNN algorithm has significantly increased. This is due to the inherent characteristics of the algorithm. When the failure rate is relatively high, equipment damage at consecutive positions may occur, resulting in zero-values in the data processed by RNN, but not in the data processed by SNN. This indicates that inserting data from a point at a greater distance is better than having no data at all. The SNN algorithm performs better than the RNN algorithm in restoring the extensively damaged data. Another point to note is that the MAEs of the SAGA deployment scheme are larger than that of the hotspot scheme when the failure rate exceeds 40%, as shown in Figure 11b,d. However, when the failure rate is below this value, the result is just the opposite. This is primarily due to sensors near the off-ramps being damaged at a predetermined failure rate, which results in the missing of data with relatively low speed values caused by ramp queuing. Using adjacent data, especially the data from locations with large spatial distances, may lead to significant errors. Figure 18 displays the absolute error between the RSE data with 60–80% sensor failure rate and the true data at 5% CV MPR. It can be seen from the figure that the values with higher errors in the SAGA scheme are more than those in the hotspot scheme. Based on the spatial distribution of sensors in Figure 19, the error value directly increases in the segment where the damaged equipment is located. Figure 20a shows the speed distribution at 5% CV MPR across the entire road. Vehicles are queued at some off-ramps due to lane-changing interference, which results in the speed on these segments is relatively low. Upon comparing Figure 18, Figure 19 and Figure 20, it can be inferred that the equipment located near the ramp in the SAGA deployment scheme is damaged when the failure rate exceeds 60%, which results in a significant discrepancy between the data processed using the RNN algorithm and the original data. Since sensors are all located on the ramps in the hotspot scheme, even if the above situation occurs, the differences between the data are not so significant. This explains why the MAEs of the SAGA deployment scheme are larger than those of the hotspot scheme when the failure rate exceeds 40%, especially 60%.

Figure 12 and Figure 13 show the distribution of MSE. Consistent with the results in Figure 10 and Figure 11, the SAGA deployment scheme outperforms the hotspot deployment scheme, and the fused data exhibits smaller errors than the non-fused data. It can also be clearly seen that the MSE of the unfused data increases with the increase in the failure rate in Figure 13b,d; however, the fluctuation range of error values is small in Figure 12b,d though they are also on a slow increasing trend. This also approves the conclusion that the data processed by the SNN algorithm is superior to the RNN algorithm. Compared with Figure 12 and Figure 13, there have been basically no other trend changes in Figure 14 and Figure 15 apart from the changes in numerical values. Relatively speaking, RMSE puts the error back into the original units, making it easier to observe and compare.

Figure 16 and Figure 17 show the distribution of MAPEs. Different from the above results, the data processed by the SNN algorithm has higher MAPEs than those processed by the RNN algorithm. This is primarily because, under conditions of a relatively high failure rate, SNN substitutes missing data with data from locations farther away from the target, leading to an overestimation or underestimation of the travel time for the segment and subsequently generating a higher MAPE value. Nevertheless, the RNN algorithm only utilizes data from the sensors closest to the target location, and the value is zero if the adjacent sensor also damaged, which makes the value of MAPE not exceed 1. The MAPEs higher than 1 pushed the overall MAPE up, which resulted in a discrepancy between the results of this indicator and that of MAE, MSE, and RMSE for unfused data.

To further observe this impact, Figure 20 plots the Absolute Percentage Errors (APEs) of the unfused data across four sampling time periods at 60% sensor failure rate when the CV MPR is 5%. As shown in the figure, peaks appear at the road segments where sensors are damaged, especially for the data processed by SNN. Take link 12–14 as an example for the explanation. The sensors numbered with 12, 13, 14, 15 (as listed in Section 4.2), which correspond to location cells of 551, 600, 608, and 665 under the hotspot scheme, and cells of 478, 483, 657, and 690 under the SAGA deployment scheme (see Table 1 and Figure 19), are all damaged at 60% sensor failure rate. The SNN uses the data collected on cells 534 and 672 for the hotspot scheme, while using the data on cells 448 and 695 for the SAGA scheme, to estimate the travel time between them. This kind of estimation data across a large span, i.e., 13.8 km and 24.7 km, leads to a significant deviation between the estimated value and the actual value. Spontaneously, it will result in large MAPEs. This explains why the MAPE in Figure 16b at 60% failure rate is so large. In this case, if possible, employing data fusion would be a better approach in improving data quality. Otherwise, it is necessary to either conduct equipment maintenance or install additional equipment.

From a comprehensive view of Figure 10, Figure 11, Figure 12, Figure 13, Figure 14, Figure 15, Figure 16 and Figure 17, the impact of sensor failure rate and the CV MPR on the MAE, MSE, and RMSE exhibits consistency, but there is a slight deviation for the MAPE. This implies that, to avoid misleading or implicit biases in the results, it is better to use indicators from different categories, such as RMSE and MAPE in this study. Another noteworthy trend is that, though the data is not fused with CV data, there are also deviations in data accuracy under different proportions of connected vehicles. Even if traffic volume remains unchanged, changes in vehicle composition can also lead to variations in traffic parameters such as travel speed and travel time. Although both the test data and the validation data come from the same driving environment, the accuracy of the data may deviate since sensor locations are predefined for every driving environment. This can also be confirmed from Figure 9.

**Figure 21:**
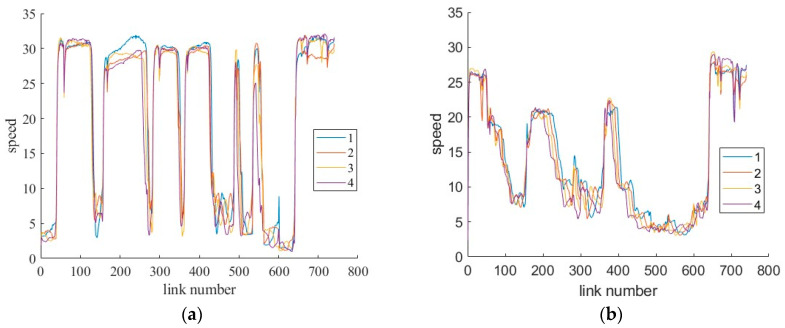
The distribution of spot speed at 5% CV MPR under two different traffic scenarios: (**a**) the initial traffic scenario; (**b**) the new traffic scenario.

To further investigate the performance of these indicators under different traffic environments, the traffic volume on the mainline has increased to 4000 veh/h and the ramp volume has also increased by 300–500 veh/h. The lane-changing parameter is adjusted (for example, the lcCooperative is changed from 0.5 to 1 for human-driving vehicles) to enhance the willingness of human-driving vehicles to collaborate during lane changes to reduce the resulting queues near ramps. The speed distribution under the two traffic scenarios is shown in Figure 21. It used the spot speed of the last four sampling periods, which are used for contrastive analysis. Compared to the speed distribution across the entire sampling interval, the data within this sample period can generally represent the overall trend, except for the data fluctuations occurring during the formation of congestion at the bottom of the curve. Vehicles are queued at some off-ramps due to lane-changing interference in the initial traffic environment, while vehicles are moving slowly without queuing on the segments with heavy traffic in the new scenario. The data error of the latter will be smaller since there is no vehicle queue. Due to / MAE, MSE, and RMSE exhibiting the same trend of change, the two error indicators, RMSE and MAPE, are selected for the following analysis.

**Figure 22:**
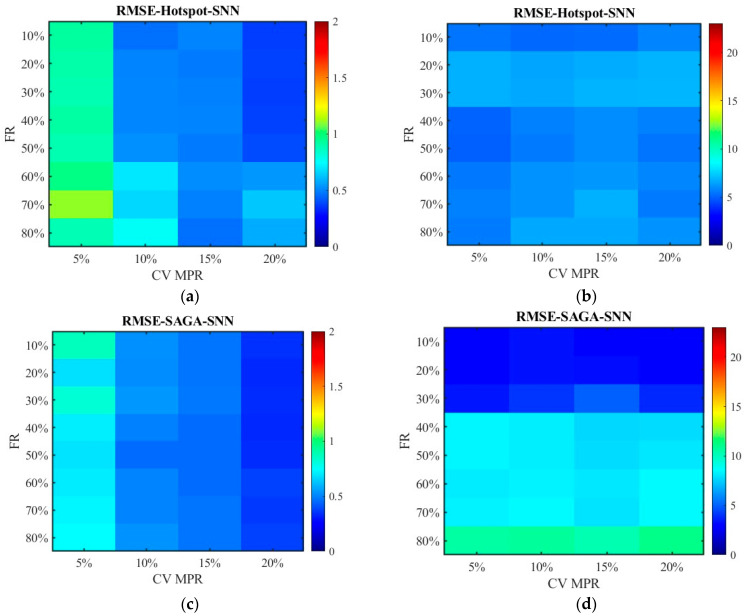
The distribution of RMSE with the SNN algorithm in the new traffic scenario: (**a**) RMSE of the fusion data under the hotspot deployment scheme; (**b**) RMSE of the pre-fusion data under the hotspot deployment scheme; (**c**) RMSE of the fusion data under the SAGA deployment scheme; (**d**) RMSE of the pre-fusion data under the SAGA deployment scheme.

**Figure 23:**
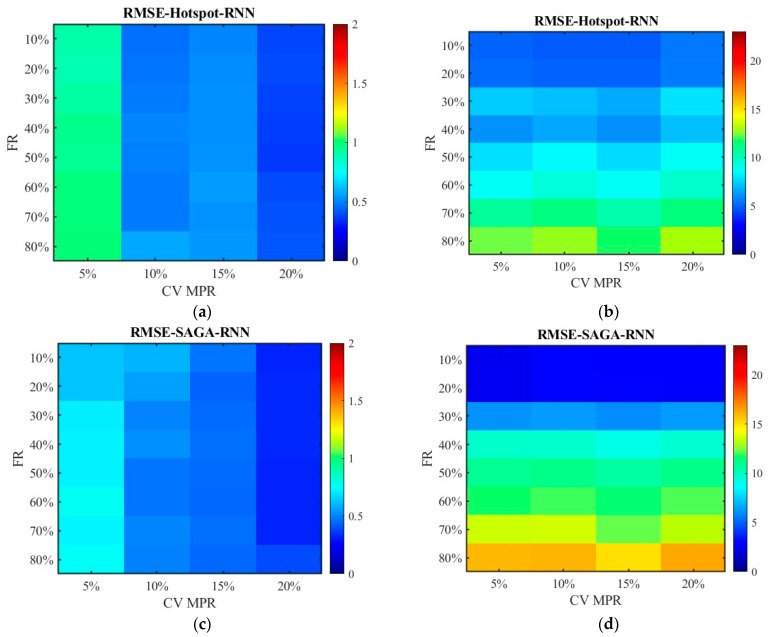
The distribution of RMSE with the RNN algorithm in the new traffic scenario: (**a**) RMSE of the fusion data under the hotspot deployment scheme; (**b**) RMSE of the pre-fusion data under the hotspot deployment scheme; (**c**) RMSE of the fusion data under the SAGA deployment scheme; (**d**) RMSE of the pre-fusion data under the SAGA deployment scheme.

Figure 22 plots the RMSE distribution for the data processed by the SNN algorithm, while Figure 23 shows the data processed by the RNN algorithm. Subgraph (a) and (b) represent the error of the data obtained under the hotspot deployment scheme, whereas subgraph (c) and (d) are under the SAGA deployment scheme. Subgraph (a) and (c) displays the RMSE of fusion data, whose values are smaller than 2. Thus, to clearly observe the trend of change, the color gamut range in heat map is set as 2. Different from them, the color gamut ranges of subfigure (c) and (d) are consistent with Figure 14 and Figure 15. The results in Figure 22 and Figure 23 are in agreement with those in Figure 14 and Figure 15, except that the overall RMSE values in Figure 22 and Figure 23 are lower. This is due to the change in road environment, which has reduced vehicle queues caused by low lane-changing cooperation and improved data quality.

**Figure 24:**
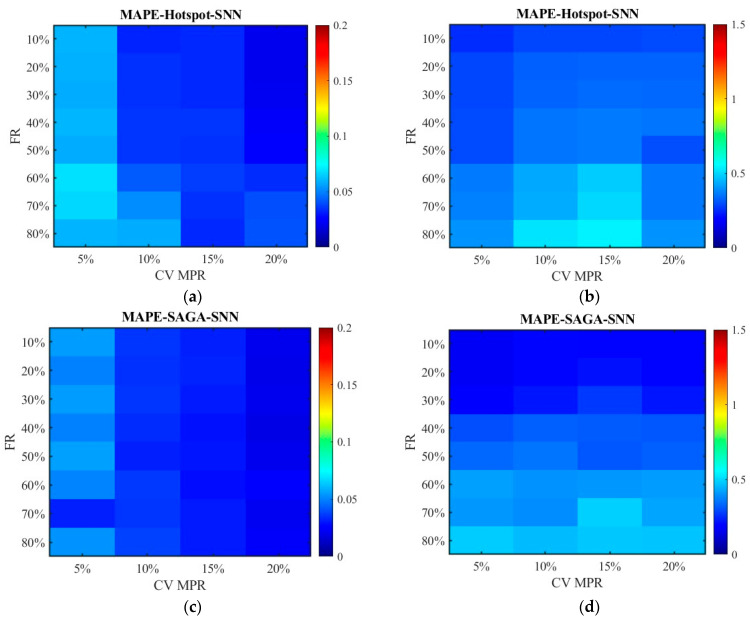
The distribution of MAPE with the SNN algorithm in the new traffic scenario: (**a**) MAPE of the fusion data under the hotspot deployment scheme; (**b**) MAPE of the pre-fusion data under the hotspot deployment scheme; (**c**) MAPE of the fusion data under the SAGA deployment scheme; (**d**) MAPE of the pre-fusion data under the SAGA deployment scheme.

**Figure 25:**
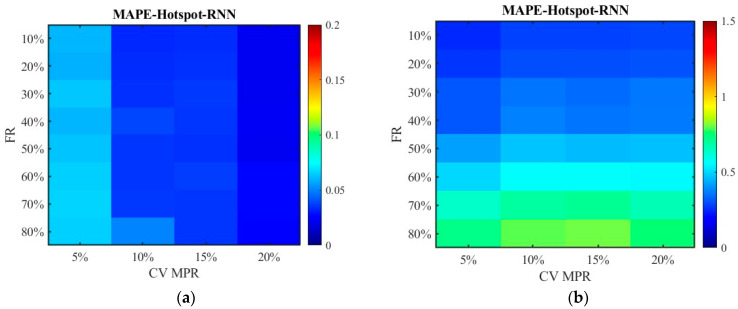

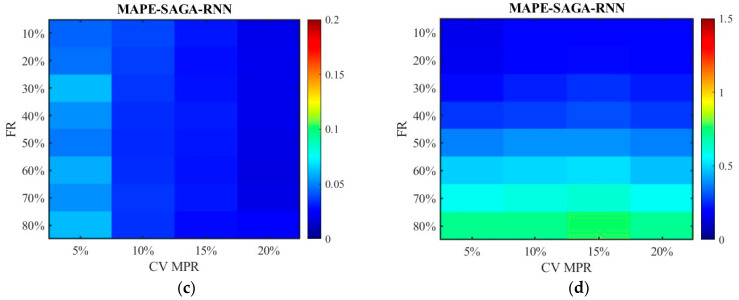
The distribution of MAPE with the RNN algorithm in the new traffic scenario: (**a**) MAPE of the fusion data under the hotspot deployment scheme; (**b**) MAPE of the pre-fusion data under the hotspot deployment scheme; (**c**) MAPE of the fusion data under the SAGA deployment scheme; (**d**) MAPE of the pre-fusion data under the SAGA deployment scheme.

Figure 24 and Figure 25 display the distribution of MAPEs. The color gamut range of the fused data is set as 0.2, while it is still 1.5 for the unfused data to be consistent with Figure 16 and Figure 17. The results in Figure 24 and Figure 25 are in line with those in Figure 16 and Figure 17, and these values are also lower. This indicates that even though the scenario has changed, the conclusion remains unchanged. Therefore, it is safe to conclude that the accuracy of the fused data is less affected by sensor failure rate but increases with the increase in CV MPR. The SNN algorithm is superior to the RNN algorithm in dealing with missing data. Under the SAGA deployment scheme, better data accuracy can be achieved. When the failure rate exceeds 40%, especially when it surpasses 60%, it is better to adopt multi-source data fusion, equipment restoration, or additional equipment deployment to improve data quality.

## 5. Conclusions

In this work, a simulation-based framework combined with data processing algorithms was proposed to explore the influence of CV MPR, sensor failure rate, and their combined effect on data accuracy. An optimal deployment method with spatial constraint, which incorporates the spatial uniformity of sensor locations into the optimization objective, was proposed to optimize the locations of RSE. Combining the global search capability of SA with the parallel search and diversity maintenance capabilities of GA, an improved SAGA incorporating spacing constraints was given to solve the optimal model. A rigid nearest neighbor algorithm and a soft nearest neighbor algorithm were addressed to handle the missing RSE data caused by sensor failure. Additionally, the BPNN algorithm was adopted to fuse the RSE data and the connected vehicle data. For comparison, other machine-learning methods, such as the LSTM model and the RF model, were also adopted to fuse the data. Subsequently, the error indicators, including MAE, MAPE, MSE, and RMSE, were calculated and compared to evaluate the data quality in different scenarios. A case study was conducted on a unidirectional four-lane freeway with sensors deployed under the hotspot deployment scheme and the optimal deployment scheme. Two distinct traffic scenarios are presented: one involves queuing at off-ramps, while the other depicts slow-moving traffic under high-volume conditions. The spot speed data and the travel speed data were acquired in SUMO by creating mixed traffic flow with CVs and human-driven vehicles. Finally, our simulation results yield several key findings as listed in Table 5: (1) The optimal deployment method is superior to the uniform method and the hotspot method in optimizing sensor deployment. Furthermore, the improved SAGA outperforms the SA algorithm and the GA in solving the proposed optimal model. (2) Data accuracy can be improved along with the increase in CV MPR. However, this improvement slows down when the MPR exceeds 15% and 60%, respectively. The MAPEs are lower than 0.05 when the MPR is 15%, and they are just lower than 0.02 when the MPR is 60%. The fused data obtained by using the BPNN algorithm substantially yielded smaller errors than the LSTM and the RF algorithm. (3) For single-source data, data accuracy decreases with the increase in sensor failure rate. However, this trend of change does not apply to fused data. The performance of the SNN method is better than the RNN method in fixing single-source missing data. However, multi-source data fusion, especially with the high-precision data, is much more effective in improving data accuracy than missing data imputation. When sensor failure rate exceeds 40%, careful selection of data-fixing methods is necessary. For sensors that are damaged at a long distance or in three or more consecutive locations, it is recommended to adopt methods such as multi-source data fusion or equipment maintenance and redeployment, rather than repairing missing data from a single source, to improve data accuracy.

In a mixed-vehicle driving environment, the functions of RSE might not only include data perception but also data communication to meet the requirements for data acquisition and data sharing between road users and infrastructure. This study only considers the impact of sensor failure on perception function, without considering the impact on the communication function. However, damage to the communication function or communication equipment will affect data acquisition for CVs. Due to the differences in functional requirements and evaluation metrics between data perception and data communication, the deployment schemes are also distinct. Under this deployment scheme, it is more appropriate to explore the impact of failures in two types of functional equipment on the mixed traffic environment. Research will be conducted on it in the next step.

## Figures and Tables

**Table 1:** Simulation parameters.

Parameters	Value
Simulator	SUMO
Road length	74 km
Number of lanes	4
Number of road segments	740
Road segment length	100 m
Vehicle speed	0–33 m/s
Volume	0–2000 veh/h for mainline, 0–800 veh/h for ramps
CV MPR	5%, 10%, 15%, 20%, 30%, 40%, 50%, 60%, 70%, 80%, 90%, 100%
Car-following model	CACC/IDM
Lane-change model	LC2013
Simulation time	7200 s
Data collection interval	300 s

**Table 2:** Cell number of sensor location under different deployment methods.

Serial Number	Uniform	Hotspot	Optimal		
			SAGA	SA	GA
1	1	1	15	1	31
2	38	39	71	31	38
3	75	59	101	68	43
4	112	157	126	78	63
5	149	168	171	96	86
6	186	282	191	100	158
7	223	299	231	114	275
8	260	362	366	208	297
9	297	370	383	218	302
10	334	487	414	226	339
11	371	534	448	298	395
12	408	551	478	364	414
13	445	600	483	403	435
14	482	608	657	437	449
15	519	665	690	580	454
16	556	672	695	605	659
17	593	709	704	635	692
18	630	717	725	658	709
19	667	723	730	664	718
20	704	740	739	734	723

**Table 3:** Initialization parameters of SAGA, SA and GA.

Parameter	Value
Initial temperature	1000
Minimum temperature	1 × 10^−3^
Temperature attenuation coefficient	0.9
Maximum number of iterations	1000
Population size	300
Crossover rate	0.8
Mutation rate	0.05

**Table 4:** Initialization parameters of BPNN.

Parameter	Value
Maximum Epochs	1000
Learning rate	0.01
Minimum error of training target	1 × 10^−5^
Train-test split ratio	20:4
Input layer	3
Hidden layer	5
Output layer	1

**Table 5:** A short summary of key findings.

Ways Affecting Data Accuracy	Scope of Research	Key Findings
Sensor allocation method	The optimal method, the uniform method, the hotspot method	The optimal method performs better.
Heuristic Algorithm	SAGA, SA, GA	The SAGA performs better.
Data fusion algorithm	BPNN, LSTM, RF	The BPNN algorithm performs better.
Data imputation algorithm	SNN, RNN	The SNN performs better.
CV MPR	5–100%	When MPR exceeds 15% and 60%, respectively, the rate of improvement in data accuracy slows down.
RSE failure rate	10–80%	The fused data is less affected by this factor; the single-source data under 40% failure rate can be improved by data imputation.

## Data Availability

Dataset available on request from the authors.

## References

[1] Mousavi S.M., Osman O.A., Lord D., Dixon K.K., Dadashova B. (2021). Investigating the safety and operational benefits of mixed traffic environments with different automated vehicle market penetration rates in the proximity of a driveway on an urban arterial. Accid. Anal. Prev..

[2] Shladover S.E. (2018). Connected and Automated Vehicle Systems: Introduction and Overview. J. Intell. Transp. Syst..

[3] Wong W., Shen S., Zhao Y., Liu H.X. (2019). On the estimation of connected vehicle penetration rate based on single-source connected vehicle data. Transp. Res. Part B Methodol..

[4] Talebpour A., Mahmassani H.S. (2016). Influence of connected and autonomous vehicles on traffic flow stability and throughput. Transp. Res. C Emerg. Technol..

[5] Martin P.T., Feng Y., Wang X. (2003). Detector Technology Evaluation.

[6] Jung Y., Oh J. (2017). Lifespan Evaluation of Traffic Detector for Automated Traffic Recorders Based on Weibull Distribution. J. Trans. Eng. A Syst..

[7] Alemazkoor N., Wang S., Meidani H. A Recursive Data-driven Model for Traffic Flow Predictions for Locations with Faulty Sensors. Proceedings of the 2018 21st International Conference on Intelligent Transportation Systems (ITSC).

[8] Salari M., Kattan L., Lam W.H.K., Ansari Esfeh M., Fu H. (2021). Modeling the effect of sensor failure on the location of counting sensors for origin-destination (OD) estimation. Transp. Res. C Emerg. Technol..

[9] Ding L., Rajapaksha P., Minerva R., Crespi N. Deep Learning for Reducing Redundancy in Madrid’s Traffic Sensor Network. Proceedings of the 2024 IEEE 49th Conference on Local Computer Networks (LCN).

[10] Zou X., Chung E., Zhou Y., Long M., Lam W.H.K. (2024). A feature extraction and deep learning approach for network traffic volume prediction considering detector reliability. Comput. Aided Civ. Infrastruct. Eng..

[11] Argote-Cabañero J., Christofa E., Skabardonis A. (2015). Connected vehicle penetration rate for estimation of arterial measures of effectiveness. Transp. Res. C Emerg. Technol..

[12] Stanek D., Huang E., Milam R.T., Wang Y.A. Measuring Autonomous Vehicle Impacts on Congested Networks Using Simulation. Proceedings of the Transportation Research Board 97th Annual Meeting.

[13] Abdeen M.A.R., Yasar A., Benaida M., Sheltami T., Zavantis D., El-Hansali Y. (2022). Evaluating the Impacts of Autonomous Vehicles’ Market Penetration on a Complex Urban Freeway during Autonomous Vehicles’ Transition Period. Sustainability.

[14] Ye L., Yamamoto T. (2019). Evaluating the impact of connected and autonomous vehicles on traffic safety. Phys. A Stat. Mech. Its Appl..

[15] Jiang Y., Wang S., Yao Z., Zhao B., Wang Y. (2021). A cellular automata model for mixed traffic flow considering the driving behavior of connected automated vehicle platoons. Phys. A Stat. Mech. Its Appl..

[16] Liu H., Kan X.D., Shladover S.E., Lu X., Ferlis R.E. (2018). Modeling impacts of cooperative adaptive cruise control on mixed traffic flow in multi-lane freeway facilities. Transp. Res. C Emerg. Technol..

[17] Xu X., Lo H.K., Chen A., Castillo E. (2016). Robust network sensor location for complete link flow observability under uncertainty. Transp. Res. B, Methodol..

[18] Salari M., Kattan L., Lam W.H.K., Lo H.P., Esfeh M.A. (2019). Optimization of traffic sensor location for complete link flow observability in traffic network considering sensor failure. Transp. Res. B Methodol..

[19] Sherali H.D., Desai J., Rakha H.A. (2006). Discrete optimization approach for locating automatic vehicle identification readers for the provision of roadway travel times. Transp. Res. B Methodol..

[20] Zhu N., Fu C., Ma S. (2018). Data-driven distributionally robust optimization approach for reliable travel-time-information-gain-oriented traffic sensor location model. Transp. Res. B Methodol..

[21] Zhan F., Wan X., Cheng Y., Ran B. (2018). Methods for multi-type sensor allocations along a freeway corridor. IEEE Intell. Transp. Syst. Mag..

[22] Sayyady F., Fathi Y., List G.F., Stone J.R. (2013). Locating traffic sensors on a highway network: Models and algorithms. Transp. Res. Rec..

[23] An K., Xie S., Ouyang Y. (2017). Reliable sensor location for object positioning and surveillance via trilateration. Transp. Res. Procedia.

[24] Fei X., Mahmassani H.S., Murray-Tuite P. (2013). Vehicular network sensor placement optimization under uncertainty. Transp. Res. C Emerg. Technol..

[25] Ban X., Herring R., Margulici J.D., Bayen A.M., Lam W., Wong S., Lo H. (2009). Optimal sensor placement for freeway travel time estimation. Transportation and Traffic Theory 2009: Golden Jubilee.

[26] Liu H.X., Danczyk A. (2009). Optimal sensor locations for freeway bottleneck identification. Comput. Aided Civ. Infrastruct. Eng..

[27] Danczyk A., Liu H.X. (2011). A mixed-integer linear program for optimizing sensor locations along freeway corridors. Transp. Res. B Methodol..

[28] Kim J., Park B., Lee J., Won J. (2011). Determining optimal sensor locations in freeway using genetic algorithm-based optimization. Eng. Appl. Artif. Intell..

[29] Zhan F., Jing P., Ran B. (2022). Infrastructure Allocation for Improving Sensing Accuracy and Connectivity Probability Based on Combination Strategy in Vehicular Networks. IEEE Trans. Intell. Transp. Syst..

[30] Kianfar J., Edara P. (2010). Optimizing freeway traffic sensor locations by clustering global-positioning-system-derived speed patterns. IEEE Trans. Intell. Transp. Syst..

[31] Barrachina J., Garrido P., Fogue M., Martinez F.J., Cano J.-C., Calafate C.T., Manzoni P. (2013). Road side unit deployment: A density-based approach. IEEE Intell. Transp. Syst. Mag..

[32] Yeferny T., Allani S. (2018). MPC: A RSUs deployment strategy for VANET. Int. J. Commun. Syst..

[33] Du Y., Wang F., Zhao C., Zhu Y., Ji Y. (2022). Quantifying the performance and optimizing the placement of roadside sensors for cooperative vehicle-infrastructure systems. IET Intell. Transport Syst..

[34] Wang Y., Zheng J. (2018). Connectivity analysis of a highway with one entry/exit and multiple roadside units. IEEE Trans. Veh. Technol..

[35] Xiao H., Liu X., Zhang Q., Chronopoulos A.T. (2021). Connectivity probability analysis for freeway vehicle scenarios in vehicular networks. Wireless Netw..

[36] Ghorai C., Banerjee I. (2018). A constrained Delaunay triangulation based RSUs deployment strategy to cover a convex region with obstacles for maximizing communications probability between V2I. Veh. Commun..

[37] Liang B., Xu X., Lu W., Wang F., Ran B. (2024). Optimizing the Deployment of Static and Mobile Roadside Units Using a Branch-and-Price Algorithm. IEEE Trans. Intell. Transp. Syst..

[38] Zeng M., He J. (2024). Deployment Optimization of Roadside Unit with Failure Probability Based on Stochastic Mixed Traffic Equilibrium. IEEE Trans. Intell. Transp. Syst..

[39] Lehsaini M., Gaouar N., Nebbou T. (2022). Efficient deployment of roadside units in vehicular networks using optimization methods. Int. J. Commun. Syst..

[40] Yu L., Zhang Z., Li J., Ma J., Wang Y. (2023). A Multi-Objective Roadside Unit Deployment Model for an Urban Vehicular Ad Hoc Network. ISPRS Int. J. Geo-Inf..

[41] Stern R.E., Cui S., Monache M.L.D., Bhadani R., Bunting M., Churchill M., Hamilton N., Haulcy R.M., Pohlmann H., Wu F. (2018). Dissipation of stop-and-go waves via control of autonomous vehicles: Field experiments. Transp. Res. C Emerg. Technol..

[42] Zheng F., Liu C., Liu X., Jabari S.E., Lu L. (2020). Analyzing the impact of automated vehicles on uncertainty and stability of the mixed traffic flow. Transp. Res. C Emerg. Technol..

[43] Gao K., Han F., Dong P., Xiong N., Du R. (2019). Connected Vehicle as a Mobile Sensor for Real Time Queue Length at Signalized Intersections. Sensors.

[44] Majstorović Ž., Miletić M., Čakija D., Dusparić I., Ivanjko E., Carić T. (2022). Impact of the Connected Vehicles Penetration Rate on the Speed Transition Matrices Accuracy. Transp. Res. Procedia.

[45] Sha D., Tang Y., Ozbay K., Gao J., Zuo F. (2025). Market Penetration Rate Optimization for Mobility Benefits of Connected Vehicles: A Bayesian Optimization Approach. Transp. Res. Rec..

[46] Yao Z., Deng H., Chen Z., He X., Ai Y., Wu Y. (2024). Linear internal stability for mixed traffic flow of CAVs with different automation levels. Phys. A Stat. Mech. Its Appl..

[47] Chin H., Okuda H., Tazaki Y., Suzuki T. Model predictive cooperative cruise control in mixed traffic. Proceedings of the IECON 2015—41st Annual Conference of the IEEE Industrial Electronics Society.

[48] Xiao G., Lee J., Jiang Q., Huang H., Abdel-Aty M., Wang L. (2021). Safety improvements by intelligent connected vehicle technologies: A meta-analysis considering market penetration rates. Accid. Anal. and Prev..

[49] Zhang W., Guhathakurta S., Fang J., Zhang G. (2015). Exploring the impact of shared autonomous vehicles on urban parking demand: An agent-based simulation approach. Sustain. Cities Soc..

[50] Yang H., Wang Z., Xie K. (2017). Impact of connected vehicles on mitigating secondary crash risk. Int. J. Trans. Sci. Technol..

[51] Papadoulis A., Quddus M., Imprialou M. (2019). Evaluating the safety impact of connected and autonomous vehicles on motorways. Accid. Anal. Prev..

[52] Shladover S.E., Su D., Lu X. (2012). Impacts of Cooperative Adaptive Cruise Control on Freeway Traffic Flow. Transp. Res. Rec..

[53] Henrickson K., Zou Y., Wang Y. (2015). Flexible and Robust Method for Missing Loop Detector Data Imputation. Transp. Res. Rec..

[54] Shang Q., Yang Z., Gao S., Tan D. (2018). An Imputation Method for Missing Traffic Data Based on FCM Optimized by PSO-SVR. J. Adv. Trans..

[55] Cottam A., Li X., Wu Y. (2025). VEMLAN: Imputing missing data for failed freeway traffic sensors. J. Intell. Trans. Syst..

[56] Li X., Ouyang Y. (2011). Reliable sensor deployment for network traffic surveillance. Transp. Res. B Methodol..

[57] Zhu N., Ma S., Zheng L. (2017). Travel time estimation oriented freeway sensor placement problem considering sensor failure. J. Intell. Trans. Syst..

[58] Sun W., Shao H., Li J., Wu T., Fainman E.Z. (2025). Multi-type traffic sensor location problem for origin–destination estimation considering spatiotemporal correlation and sensor failure. Transp. Res. C Emerg. Technol..

[59] Liang B., Wang F., Ran B. (2024). Optimizing Roadside Unit Deployment in VANETs: A Study on Consideration of Failure. IEEE Trans. Intell. Transp. Syst..

[60] Chaudhuri P., Martin P.T., Stevanovic A.Z., Zhu C. (2010). The effects of detector spacing on travel time prediction on freeways. World Acad. Sci. Eng. Technol..

[61] Yang J., Peng Z., Lin L. (2021). Real-time spatiotemporal prediction and imputation of traffic status based on LSTM and Graph Laplacian regularized matrix factorization. Transp. Res. C Emerg. Technol..

[62] Danczyk A., Di X., Liu H.X. (2016). A probabilistic optimization model for allocating freeway sensors. Transp. Res. C Emerg. Technol..

[63] Du Y., Sun C.P. (2022). A novel interpretable model of bathtub hazard rate based on system hierarchy. Reliab. Eng. Syst. Saf..

